# Lipid Production in Cultivable Filamentous Fungi Isolated from Antarctic Soils: A Comprehensive Study

**DOI:** 10.3390/microorganisms13030504

**Published:** 2025-02-25

**Authors:** Victor Gallardo, Jéssica Costa, Marcela Sepúlveda, Yasna Cayún, Christian Santander, Excequel Ponce, Juliana Bittencourt, César Arriagada, Javiera Soto, Romina Pedreschi, Vania Aparecida Vicente, Pablo Cornejo, Cledir Santos

**Affiliations:** 1 Doctoral Program in Science of Natural Resources, Universidad de La Frontera, Temuco 4811230, Chile; v.gallardo04@ufromail.cl (V.G.); m.sepulveda23@ufromail.cl (M.S.); 2 Postgraduate Program in Biotechnology, Federal University of Technology-Paraná, Ponta Grossa 84017-220, Brazil; julianavitoria@utfpr.edu.br (J.B.); vaniava63@gmail.com (V.A.V.); 3 Departamento de Biologia, Instituto de Ciências Biológicas—ICB, Universidade Federal do Amazonas, Manaus 69080-900, Brazil; jessicacosta@ufam.edu.br; 4 Department of Chemical Science and Natural Resources, Universidad de La Frontera, Temuco 4811230, Chile; yasna.cayun@ufrontera.cl (Y.C.); c.santander01@ufromail.cl (C.S.); 5 Postgraduate Program in Microbiology, Parasitology and Pathology, Federal University of Parana, Curitiba 80060-000, Brazil; 6 Escuela de Agronomía, Facultad de Ciencias Agronómicas y de los Alimentos, Pontificia Universidad Católica de Valparaíso, Quillota 2340025, Chile; excequel.p.g@gmail.com (E.P.); romina.pedreschi@pucv.cl (R.P.); 7 Laboratorio Biorremediación, Departamento de Ciencias Forestales, Facultad de Ciencias Agropecuarias y Medioambiente, Universidad de La Frontera, Temuco 4811230, Chile; cesar.arriagada@ufrontera.cl (C.A.); javiera.soto@ufrontera.cl (J.S.); 8 Millennium Institute Center for Genome Regulation (CRG), Santiago 8331150, Chile; 9 Postgraduate Program in Bioprocess Engineering and Biotechnology, Federal University of Parana, Curitiba 84017-220, Brazil; 10 Plant Stress Physiology Laboratory, Centro de Estudios Avanzados en Fruticultura (CEAF), Rengo 2940000, Chile; 11 Centro Tecnológico de Suelos y Cultivos (CTSyC), Facultad de Ciencias Agrarias, Universidad de Talca, Talca 3460000, Chile; 12 Centro Regional de Investigación e Innovación para la Sostenibilidad de la Agricultura y los Territorios Rurales, CERES, La Palma, Quillota 2260000, Chile

**Keywords:** oleaginous fungi, lipid extraction, bioactive lipids

## Abstract

Antarctic soil represents an important reservoir of filamentous fungi (FF) species with the ability to produce novel bioactive lipids. However, the lipid extraction method is still a bottleneck. The objective of the present work was to isolate and identify cultivable FF from Antarctic soils, to assess the most effective methods for fatty acid (FA) extraction, and to characterise the obtained lipids. A total of 18 fungal strains belonging to the *Botrytis*, *Cladosporium*, *Cylindrobasidium*, *Mortierella*, *Penicillium*, *Pseudogymnoascus*, and *Talaromyces* genera and the Melanommataceae family were isolated and identified. The Folch, Bligh and Dyer, and Lewis extraction methods were assessed, and methyl esters of FA (FAMEs) were obtained. The Lewis method was the best in recovering FAMEs from fungal biomass. A total of 17 FAs were identified, and their chemical compositions varied depending on fungal species and strain. Oleic, linoleic, stearic, and palmitic acids were predominant for all fungal strains in the three assessed methods. Among the analysed strains, *Cylindrobasidium eucalypti*, *Penicillium miczynskii*, *P. virgatum*, and *Pseudogymnoascus pannorum* produced high amounts of FA. This suggests that the soils of Antarctica Bay, as well as harbouring known oleaginous fungi, are also an important source of oleaginous filamentous fungi that remain poorly analysed.

## 1. Introduction

The Antarctic territory offers a range of challenging conditions, such as low temperatures, dryness, low nutrient content, high salinity, a high UV incidence, and freeze–thaw cycles [1,2,3]. This environment represents a gateway to studies on the taxonomy, ecology, and biotechnology of organisms under extreme conditions. Fungi are ubiquitous and diverse organisms in Antarctica and have been described as growing on different substrates, such as plants, soil, rocks, ice, snow, and animals [1,2,3]. To survive in such extreme conditions, fungi might display unusual biochemical pathways able to generate specific or novel compounds with medical and biotechnological relevance. Before accessing the fungal biotechnological potential, knowing the fungal species is mandatory [4,5].

As an adaptation, saturated and unsaturated fatty acids (FAs) have an important role in regulating biotic and abiotic stresses in Antarctic fungi [6,7]. The diversity and amounts of FA can vary from one to another organism, influenced by developmental stages, nutrition, and environmental conditions [6]. Due to their ability to accumulate lipids ranging from 20 to 70% of their dry cell weight, strains of the *Aspergillus*, *Cunninghamella*, *Fusarium*, *Mortierella*, *Mucor*, and *Penicillium* genera are known as oleaginous fungi [8,9,10,11,12].

Antarctic fungi are a rich source of new compounds [13,14]. However, until now, the potential of Antarctic fungi as a source of FAs and their application in biotechnological processes has been underexplored. To achieve this target, establishing and optimising the process of FA extraction is crucial, especially for fungal strains obtained from pristine natural environments that have been minimally investigated.

Although the Bloor [15] method has widely been used for lipid extraction, it is a time-consuming method with several steps involving the use of different organic solvents (e.g., ethanol, ether, chloroform, and petroleum). The Folch, Bligh and Dyer, and Lewis methods have been used as alternative approaches. The Folch method can be used on different types of samples, while the Bligh and Dyer method is compatible only with shorter samples [9,16]. Overall, the Lewis method is, by far, the simplest and most effective method to extract lipids from different sources. The method has the advantage of avoiding lipid degradation and allowing for subsequent use for analysis in a single step [17]. The objective of the present work was to isolate and identify cultivable filamentous fungi (FF) from Antarctic soils, to assess the most effective method for lipid extraction, and to characterise the lipids extracted from fungal cell membranes.

## 2. Materials and Methods

### 2.1. Soil Sampling

**Figure 1:**
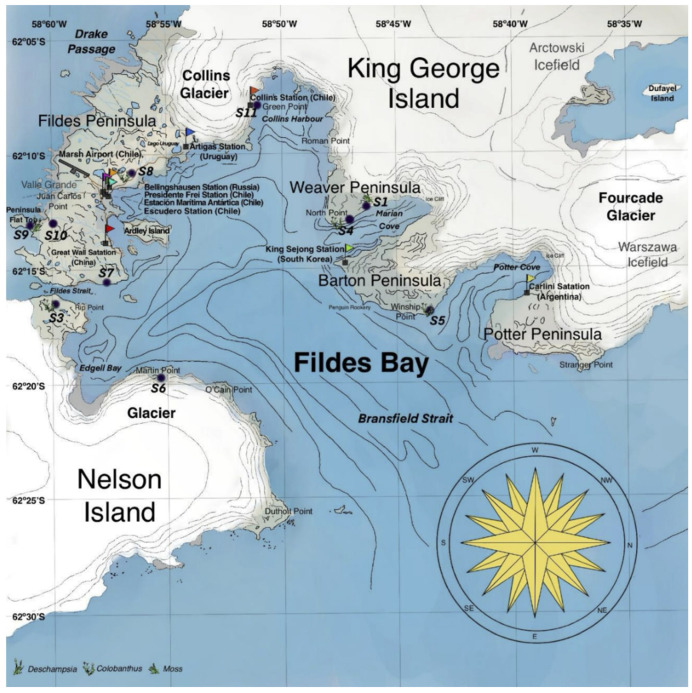
Map of Fildes Bay with the geographic distribution of sampling points used in the present study. Adapted from Gallardo et al. [3].

A total of 10 soil samples were obtained during the 58th Chilean Antarctic Expedition, from January to February 2022. Soil samples were collected from King Jorge Island—S1 (62°12′20.40″ S, 58°46′20.16″ W), S4 (62°12′39.71″ S, 58°47′20.07″ W), S5 (62°14′24.90″ S, 58°43′29.86″ W), S7 (62°13′51.1″ S, 58°58′04.6″ W), S8 (62°11′29.12″ S, 58°56′42.31″ W) S9 (62°12′39.82″ S, 59°01′08.18″ W), S10 (62°12′26.9″ S, 59°00′14.1″ W), and S11 (62°10′09.7″ S, 58°51′05.2″ W)—and from Nelson Island—S3 (62°14′6.59″ S, 59°0′6.19″ W) and S6 (62°15′39.8″ S, 58°55′42.9″ W) (Figure 1). Soil samples were collected using a transect of 4 × 25 m at a depth of 00–20 cm [18]. A total of ten samples taken along the transect were combined to form a single composed sample. Each sample (0.5–1 kg) was collected with a sterile spoon into appropriately labelled airtight containers with the name and the coordinates of the collection sector. Then, all samples were stored in a cooler (at approximately 4 °C) until mycological isolation at the Laboratory of Fungal Chemistry located at Universidad de la Frontera.

### 2.2. Soil Chemical Characterisation

Chemical analysis of soil was performed according to the methodology described by Chávez [19]. Briefly, soil pH was measured using a potentiometer. To obtain the soil pH, a glass electrode was placed into a suspension composed of soil and deionised water (2:5, *w*/*v*). Available Phosphorus (P) was determined in a 0.5 M NaHCO_3_ solution (pH 8.5) [20]. Sodium (Na), Magnesium (Mg), Potassium (K), and Calcium (Ca) were quantified by atomic absorption spectrophotometry (Perkin Elmer, Waltham, MA, USA, mod. PinAAcle 500). The percentage of Organic Carbon (%OC) was determined by inverse titration by oxidation with K_2_Cr_2_O_7_ in acidic medium [21]. The percentage of Nitrogen (%Nt) was determined based on the Kjeldahl method [22].

### 2.3. Fungi Isolation

Soil samples (30 g) were suspended in 300 mL of distilled water, and 3 serial dilutions (1:10, *v*/*v*) were prepared. One hundred microliters of stock solution and serial dilutions were inoculated on Petri plates containing each of the following media: potato dextrose agar (PDA, 3 g potato extract, 20 g glucose, 15 g agar, and 0.1 g chloramphenicol), 18% dichloran glycerol agar (DG18, 18% glycerol, 1 g KH_2_PO_4_, 0.5 g MgSO_4_ · 7H_2_O, 5 g mycological peptone, 0.002 g dichloran, 0.1 g chloramphenicol, 15 g agar, and 10 g glucose), and dichloran Rose Bengal chloramphenicol agar (DRBC, 1 g KH_2_PO_4_, 0.5 g MgSO_4_ · 7H_2_O, 5 g mycological peptone, 0.002 g dichloran, 0.1 g chloramphenicol, 15 g agar, 10 g glucose, and 0.025 g Rose Bengal [18]. Petri plates were incubated at 10 °C for 30 days in the dark. To purify fungal isolates, fragments of fungal colonies were transferred onto Petri plates containing PDA medium and incubated at 10 °C for 30 days.

### 2.4. Fungal Identification Based on Morphological Analysis

For fungal morphological identification, all strains were subcultured on PDA at 4 °C for 7–28 days in the dark. After growth, macro- and microscopic traits of strains were registered using a camera (QIMAGING, Surrey, Canada, Micropublisher, 3.3 RTV) and an IS.1153 PLPHi Euromex Microscope (Euromex, Arnhem, The Netherlands), respectively. Fungal strains were identified at the genus level based on macro- and micro-morphological traits with appropriate keys [18,23,24,25,26,27]. All fungal strains isolated in the present study were deposited at the Bank of Microbiological Resources of the Universidad de La Frontera (Temuco, Chile), which is registered with the World Federation for Culture Collection under number WDCM 1283.

### 2.5. Fungal Identification Based on Molecular Biology Analysis

Genomic DNA of each fungal strains was extracted using the SDS method as previously described by Rodrigues et al. [28]. The ITS1-5.8S-ITS2 rDNA region was amplified using the ITS1 (5′-TCC GTA GGT GAA CCT GCG G-3′) and ITS4 (5′-TCC TCC GCT TAT TGA TAT GCC-3′) primers designed by White et al. [29]. For ITS reaction, 25 μL of Taq DNA polymerase Master Mix 2× (VWR Life Science, Radnor, PA, USA), 2 μL of primer ITS1 10 mM, 2 μL of primer ITS4 10 mM, 2 μL of genomic DNA, and 19 μL of distilled water free of DNases were combined in a final volume of 50 μL. The PCR parameters used in the thermal cycler were 94 °C for 3 min, 35 cycles of 94 °C for 1 min, 55 °C for 1 min, 72 °C for 1 min, and a final extension at 72 °C for 5 min. PCR parameters used in the thermal cycler were 95 °C for 3 min, 35 cycles of 95 °C for 1 min, 56 °C for 45 s, 72 °C for 90 s, and a final extension at 72 °C for 10 min.

Bands were visualised on 1% (*w*/*v*) agarose gel supplemented with SYBR^®^ Safe DNA Gel Stain (Invitrogen, Carlsbad, CA, USA) as a staining element and NZYDNA ladder III as a DNA molecular weight marker. PCR products were cleaned using an ExoSAP kit (ExoSAP, Lisbon, Portugal) according to instructions of the manufacturer. The nucleotide sequences were obtained by Stab Vida Portugal Laboratories. Each sequence was manually edited using Genius Prime analysis software (https://www.geneious.com, accessed on 20 April 2024) and compared with the GenBank database using the Basic Local Alignment Search Tool (BLAST, https://blast.ncbi.nlm.nih.gov/, accessed on 20 April 2024). Sequences were aligned on the Clustal Omega web server from EMBL-EBI (https://www.ebi.ac.uk/jdispatcher/msa/clustalo, accessed on 20 April 2024) [30]. Phylogenetic analyses were performed by determining the best-fit substitution model using the maximum likelihood method on the IQ-TREE web server [31]. Trees were generated and modified using iTOL v5 [32]. The trees were created according to the data obtained in phylogenetic trees available in the literature [33,34,35,36,37,38,39,40,41] and the ITS sequences available in the GenBank database (Table S6).

### 2.6. Fungal Cultivation for Biomass Production

For fungal biomass production, spores and mycelia of strains grown on PDA plates at 10 °C for 10 days were harvested from the agar cultures using a sterile saline solution. A standard liquid medium (SL, 30 g glucose, 5 g yeast extract, 7 g KH_2_PO_4_, 2 g Na_2_HPO_4_, 1.5 g MgSO_4_ · 7H_2_O, 0.1 g CaCl_2_ · 2H_2_O, 0.008 g FeCl_3_·6H_2_O, 0.001 g ZnSO_4_ · 7H_2_O, 0.0001 g CoSO_4_ · 7H_2_O, 0.0001 g CuSO_4_ · 5H_2_O, and 0.0001 g MnSO_4_ 5H_2_O) was prepared according to the protocol described by Kosa et al. [42]. A number of conidia or mycelia concentration was transferred into Erlenmeyer flasks (250 mL) containing 150 mL of SL medium, and samples were incubated at 100 rpm for 10 days at 10 °C. Fungal biomasses were harvested, frozen at −80 °C, and freeze-dried using an L101 (Liobras, São Carlos, SP, Brazil) for five days at −55 °C and 0.01 mbar pressure.

### 2.7. Lipid Extraction from Fungal Biomass

For each fungal strain, 250 mg of freeze-dried biomass was weighted, and lipid extractions were performed using the following 3 methods:

#### 2.7.1. Folch Extraction Method

For the Folch method [43], each freeze-dried biomass was transferred into a 15 mL polypropylene tube and soaked with deionised water to create a moisture content of 80% (*w*/*w*). For each 250 mg of freeze-dried biomass, 0.2 mL of water was added. Samples were homogenised with 5 mL of chloroform:methanol solution (2:1, *v*/*v*) using a thin glass bar. Neat methanol (0.3 mL) was added and homogenised for 30 s using a vortex.

A tube (tube A) containing biomass and a solvent phase was centrifuged at 2800 g and 20 °C for 10 min. The solvent phase was transferred into a new polypropylene tube (tube B). Neat chloroform (0.6 mL) was added to the tube containing the biomass (tube A), and the sample was centrifuged. The chloroform phase was recovered from tube A and added to tube B. The solvent phase in tube B was washed with 0.2× its volume with a KCl aqueous solution (0.88%, *v*/*v*). The sample was centrifuged, and the lower phase (chloroform + lipids) was recovered in a new polypropylene tube. Residual water was removed by the addition of anhydrous K_2_HPO_4_ to the tube. In order to remove the salt, the solvent phase was transferred into a new tube. The sample was evaporated using a rotary evaporator (DLAB, RE100-Pro, Beijing, China) at 80 rpm, 60 °C, and 600 mbar until the elimination of the solvent.

Lipid transesterification was performed as described by Singh et al. [44]. Briefly, 2 mL of hexane and 1 mL of 2 M methanolic potassium hydroxide solution were added to the lipid crude extract and vortexed for 30 s. The sample was left at 70 °C for 20 min, then cooled down to room temperature (20 °C). Then, hydrochloric acid (1 M, 1.2 mL) was added, followed by the addition of 1 mL of hexane. The sample was vortexed for 30 s and left at room temperature until the separation of phases (approximately 15 min). Residual water was removed by the addition of a small amount of K_2_HPO_4_ to the tube. The upper phase, which was composed of hexane and fatty acid methyl esters (FAMEs), was recovered and stored in an amber vial at −80 °C until further analysis. Yields were determined based on the difference in weight between the dry biomass and total lipid extract [8].

#### 2.7.2. Bligh and Dyer Extraction Method

For the Bligh and Dyer method [45], freeze-dried biomass was transferred into a 15 mL polypropylene tube and soaked in deionised water to create a moisture content of 80% (*w*/*w*). For each 250 mg of freeze-dried biomass, 0.2 mL of water was added. The sample was homogenised with 0.75 mL of methanol:chloroform solution (2:1, *v*/*v*) using a thin glass rod. Initial solvent:freeze-dried sample ratio was 3:1 (*v*/*w*). To generate a final solvent:freeze-dried sample ratio of 4:1 (*v*/*w*), chloroform (0.25 mL) was added, and to create a chloroform:methanol:water ratio of 2:2:1.8 (*v*/*v*/*v*), deionised water (0.25 mL) was added to the sample.

A tube (tube A) containing biomass and a solvent phase was centrifuged at 2800 g and 20 °C for 10 min. The solvent phase was transferred into a new polypropylene tube (tube B). The sample was centrifuged to separate the methanol–water and chloroform phases. The lower phase (chloroform + lipids) was recovered in a new polypropylene tube. Residual water was removed by the addition of anhydrous K_2_HPO_4_ to the tube. In order to remove the salt, the solvent phase was transferred into a new tube. The sample was evaporated using a rotary evaporator (DLAB, RE100-Pro, China) at 80 rpm, 60 °C, and 600 mbar until solvent elimination. Transesterification was performed according to the methodology described by Singh et al. [44], as shown above. Yields were determined based on the difference in weight between the dry biomass and total lipid extract [8].

#### 2.7.3. Lewis Direct Transesterification Extraction

For the Lewis method [17], a freeze-dried biomass sample (250 mg) was transferred into a 50 mL polypropylene tube, and 25 mL of methanol:hydrochloric acid:chloroform (10:1:1 *v*/*v*/*v*) was added. Sample was vortexed for 30 s, then kept at 90 °C for 1 h. The sample was then held at room temperature to cool down. Deionised water (5 mL) and 10 mL n-hexane:chloroform (4:1, *v*/*v*) were added. The sample was vortexed for 30 s and centrifuged (2800 g, 20 °C for 10 min). The upper phase was recovered with a Pasteur pipette and transferred into a 50 mL polypropylene tube. The extraction process was repeated three times. Residual water was removed by the addition of a small quantity of anhydrous K_2_HPO_4_. The solvent phase was transferred into a new tube for salt removal. The sample was evaporated using a rotary evaporator (DLAB, RE100-Pro, China) at 80 rpm, 60 °C, and 600 mbar until the evaporation of the solvent. FAMEs were stored at −80 °C in neat hexane until further analysis. Yields were determined based on the difference in weight between the dry biomass and total lipid extract [8].

### 2.8. Monitoring Lipid Extraction with Infrared Spectroscopy

Lipid extraction was monitored using an Attenuated Total Reflectance–Fourier Transform Infrared (ATR-FT-IR) spectrometer (Cary 630 FT-IR, Aligent, Santa Clara, CA, USA). Infrared spectra were obtained at the mid-IR wavelength. The biomass of each freeze dyer (before extraction, BE) and the residual biomass (after extraction, AE) were directly exposed on the glass of the infrared spectrometer reader. Spectra were obtained in the 4000–600 cm^−1^ region with a resolution of 4 cm^−1^. The spectral regions from 3100 to 2800 cm^−1^ and from 1800 to 700 cm^−1^ were analysed for the prediction of the lipid profile [8,9] using Origin Pro 8 (Microcal Software, Northampton, MA, USA).

### 2.9. Chemical Characterisation and Quantification of Lipids by GC-MS and GC-FID

A gas chromatograph (GC) coupled with a mass spectrometer (MS) and a GC coupled with a flame ionization detection (FID) system (Agilent, Santa Clara, CA, USA) were used. The GC-MS/GC-FID was equipped with an Agilent 7890B PAL3 autosampler and a 5977A MS mass spectrometer with an EI350 electron impact ionization source. The column used was a SUPELCO SPTM-2560 (100 m × 0.25 mm × 0.2 µm). FA identification and quantification were assessed based on the presence of FAMEs in the total lipid extract. The correlation of FAME/FA was 1:1. FAME identification and quantification were carried out by GC-MS and GC-FID, respectively [46].

For GC-MS, the follow conditions were followed: an injection volume of 1 µL; injector: 220 °C; interphase: 280 °C; injection mode: split (1:50); initial temperature of oven: 80 °C; temperature ramp: 225–25 °C min^−1^, 20 min. The MS detector conditions were set as follows: helium flux: 1.0 mL/min; ionization source: 230 °C; quadrupole: 150 °C; Modo SCAN: 50–600 *m*/*z* at 2.66 cycles/second. Total quantification was carried out using an FID detector. The FID detector conditions were set as follows: helium flux: 1.6 mL/min; detector: 225 °C; air: 400 mL/min; hydrogen: 30 mL/min. For identification, a standard CRM45885 Supelco 37 kit (Merck KGaA, Darmstadt, Germany) was used, and for quantification, standards C18:0 (Stearic acid, S5376 Sigma, Saint Louis, MI, USA), C18:1 (Oleic acid, 311111 Sigma), and C18:3 (α-Linolenic acid, 62200 Sigma) were used.

### 2.10. Statistical Analysis

In the present study, the statistical analysis was developed using R Studio software (R version 4.3.1). Non-parametric analysis of the data was performed using the Kruskal–Wallis test and the Dunn post hoc test with an α value of 0.05. For the analysis, both the Stats version 4.3.1 package and the FSA version 0.9.5 package were applied. Heat maps and dendrograms were obtained using the Pheatmap version 1.0.12 package, the base version 4.3.1 package, and the Grdevices version 4.3.1 package. As clustering_method was set as “complete”, clustering_distance_cols = “canberra” and clustering_distance_rows = “binary”.

## 3. Results and Discussion

### 3.1. Soil Chemical Analysis

Soil samples from Fildes Bay presented values of organic carbon (%OC) (1.7–11.9%) and pH (6.2–7.4) similar to those of mineral soils (%OC = c.a. 13%, pH = 6.4–7.6), as previously reported by Bockheim [47] and Rosa [48]. Among samples, S7 presented the highest C/N ratio (c.a. 55), while sample S5 presented the lowest C/N ratio (c.a. 2). Notably, samples S8, S4, and S5 presented the highest Phosphorus (P) values of c.a. 187, 274, and 371 mg/kg, respectively. Sample S1 showed the lowest sodium (Na) value (c.a. 74 mg/kg), while sample S10 showed the highest Na value (c.a. 726 mg/kg) (Table 1).

Soil samples S1, S3, S4, S5, S8, and S9 were collected from soils with herbaceous vegetation of the *Deschampsia* and *Colobanthus* genera or moss and presented pH values below 7.0. In contrast, the soil samples S6, S7, S10, and S11 were taken from soils free of vegetation and presented pH values over 7.0 (Table 1). The presence or absence of vegetation influenced the variation of pH in this set of soils [49,50]. Nevertheless, the variability of chemical characteristics can be affected by other abiotic and/or biotic factors.

The chemical properties of soil depend on the life cycle of the available flora and fauna. A higher nutrient content (e.g., nitrogen) also suggests a greater impact of fauna through the deposition of animal excreta, such as guano [51]. Therefore, the presence of flora influences soil acidification, allowing for the availability of nutrients and the metabolic activity of soil microorganisms [52]. Despite the above, both the physical properties of mineral soils and extreme environmental conditions influence the retention and mobilization of nutrients. As a matter of consequence, nutrient inputs are leached or displaced to other areas, depriving the soil of nutrients [47,48]. Consequently, these characteristics could help explain the differences in C/N ratios and nutrients found among the soils and how these influence the development of life in Antarctic soils.

### 3.2. Isolation and Identification of Fungal Strains

A set of c.a. 1800 FF strains was isolated from Antarctic soils. The isolates were grouped on the basis of macro- and micro-morphological characteristics [3]. A random selection of 10% of each group was made. Subsequently, a set of FF strains was selected from each group. Each final set of FF corresponded to 10% of each taxonomic group, resulting in a total of 18 FF strains, as presented in Table 2.

Strains belonging to the *Cladosporium*, *Penicillium*, and *Pseudogymnoascus* genera and one strain belonging to the Dothideomycete class were classified. According to molecular classification using ITS, the aforementioned identifications were confirmed at the genus level. In contrast, based on molecular information, the strain belonging to the Dothideomycete class (UFRO22.418) was reclassified into the Melanomataceae family. Some other strains were reclassified by molecular biology into the *Botrytis*, *Cylindrobasidium*, *Talaromyces*, and *Mortierella* genera (Mucoromycota division). Overall, phylogenetic analysis revealed that the total dataset included 8 genera and 14 different fungi species (Table 2). Phylogenetic trees are presented in Figures S1–S8.

The *Cladosporium*, *Penicillium*, *Pseudogymnoascus*, and *Mortierella* genera have been reported as the most cosmopolitan in Antarctic soil samples [48,53,54]. In contrast, the *Botrytis*, *Cladosporium*, and *Talaromyces* genera are less frequent in Antarctic soils [55,56]. The Melanomataceae family encompasses species that are usually isolated from permafrost (e.g., soil or underwater sediment), which continuously remains below 0 °C (Figure S8). Fungi belonging to the Melanommataceae family (e.g., *Alpinaria rhododendri*) can be found in root and rhizosphere samples [57].

### 3.3. Lipid Extraction and FAME Production

Establishing fast and efficient protocols for extracting lipids from fungal biomass is a fundamental step in (i) understanding the lipid composition, (ii) selecting the most promising lipid-producing strains, and (iii) optimising fungal growth conditions to obtain a higher lipid yield. Lipid extraction steps are even more important when the studied microorganisms come from pristine environments that have been minimally or never studied before with regard to lipid composition and efficient extraction protocols [9].

In the present study, the Bligh and Dyer, Folch, and Lewis methods were applied to extract lipids from strains of the *Botrytis*, *Cladosporium*, *Mortierella*, *Penicillium*, *Pseudogymnoascus*, and *Talaromyces* genera and of the Melanommataceae family. To compare the overall extraction performance of the Bligh and Dyer, Folch, and Lewis methods, the total lipid extract value (TLE%) was calculated. The TLE% values corresponded to the total lipid yield obtained by each method. The best results for TLE% were observed for the Folch and Lewis methods.

According to statistical analysis, no significant differences between the Folch and Lewis methods were observed (Table 3). The effectiveness of lipid extraction in each fungal strain was assessed (Table S1). According to the obtained data, the Bligh and Dyer, Folch, and Lewis methods led to total lipid extract yields in the ranges of 0.84–15.79%, 2.01–28.15%, and 3.28–17.53%, respectively.

The effectiveness of FAME production among the three methods was assessed. The Lewis method was the best in recovering FAMEs from fungal biomass. Significant statistical differences were observed (*p*-value < 0.05) (Table 3). The effectiveness of FAME production in each fungal strain was also assessed. The Lewis method was the best, yielding FAME values ranging from 20.7 to 79.8%, while under the Folch and Bligh and Dyer methods, the FAME percentages were 1.25–37.86% and 5.33–55.30%, respectively (Table 3). Unlike the other two methods, in the Lewis method, HCl is added to extract and derivatise lipid molecules into FAMEs. This procedure is applied in a single step, avoiding the loss of lipid molecules.

The lowest lipid extraction yields and FAME abundance obtained with the Bligh and Dyer method can be explained by the characteristics of the extraction itself. According to Iverson et al. [58] and Forfang et al. [9], the Bligh and Dyer method can lead to an underestimation of the lipid content and FAME abundance. The small amount of solvent used is not enough to drag the total lipid towards the chloroform phase, translating into a small amount of FA that can be converted into FAMEs. Selecting the best strains, fungal growth parameters, and the right extraction method are key points that impact FAME yields.

In the present study, for the UFRO22.77 and UFRO22.262 *Botrytis cinerea* strains, the Lewis method led to the best FAME yields of 45.73% and 20.70%, respectively (Table 4). In previous studies, TLE% values ranging from 2.70 to 11.90% were obtained from the lipid composition of *B. cinerea* strains [59]. These variations in TLE% may not only be subject to the strains but also to the fungal culture parameters [6]. A TLE% yield of 6.7% for *B. cinerea* with the Folch method has also been previously reported. In this case, a FAME yield of 3.1% for *B. cinerea* with the Bligh and Dyer method was obtained [60,61]. Li et al. [62] analysed lipid composition in the *Cladosporium* genus and obtained a TLE% of 16.5% with the Bligh and Dyer method, while a FAME yield ranging from 16 to 34% with the Folch method was obtained for *Cladosporium herbarum* [63].

In the present study, lower FAME yields were obtained using both the Bligh and Dyer and Folch methods. The overall abundance of FAMEs obtained with the Folch and Bligh and Dyer methods ranged from 6.8 to 9.74% and 5.33 to 12.29%, respectively. Meanwhile, with the Lewis method, the FAME yield was over 40% for the analysed *Cladosporium* strains (*C. herbarum* UFRO22.307, *C. perangustum* UFRO22.53, and *C. varians* UFRO22.551).

The *Mortierella* genus comprises the major lipid producers’ fungal group. According to previous data [9], for the *Mortierella* genus, FAME yields ranging from 0.68 to 12.81%, 1.3 to 21.2%, and 25.8 to 28.9% were obtained with the Bligh and Dyer, Folch, and Lewis methods, respectively.

In the present study, FAMEs obtained from *Mortierella* strains with the Lewis method showed a yield at least twice as high as those obtained with the Folch and Bligh and Dyer methods. For *Mortierella antartica* UFRO22.73, the Lewis method was 40 times more efficient than the Folch method. In this case, under the Lewis method, the FAME yield was 51.93%, while under the Folch method, the FAME yield was 1.25%. For *M. turficola* UFRO22.261, the FAME yield obtained with the Lewis method was 60.34%, which was five times higher than that obtained with the Bligh and Dyer method, which yielded 11.75% FAMEs (Table 4).

Previous studies have additionally assessed the production of lipids by different species of the *Penicillium* genus, emphasising the ability of *P. atrovenetum*, *P. brevicompactum*, *P. chrysogenum*, *P. lilacinurn*, *P. citrinum*, and *P. funiculosum* to produce lipids [64,65,66,67,68,69]. Despite this, there is no information available about methods for lipid extraction and FAME recovery from species belonging to the *Penicillium* genus.

In the present study, *Penicillium miczynskii* UFRO22.569 and *P. virgatum* UFRO22.251 were assessed for their abilities to produce lipids. According to the data obtained herein, the Lewis method was the best method for lipid extraction and FAME recovery, with yields of 65.85 and 51.01% for *P. miczynskii* UFRO22.569 and *P. virgatum* UFRO22.25, respectively. FAME yields obtained by the Bligh and Dyer method were 28.62% and 6.52% for *P. miczynskii* UFRO22.569 and *P. virgatum* UFRO22.251, respectively, while FAME yields obtained by the Folch method were 6.52 and 7.90% for *P. miczynskii* UFRO22.569 and *P. virgatum* UFRO22.251, respectively.

The Melanommataceae family is the largest family of ascomycetes, encompassing a highly diverse range of fungi, including phytopathogenic, endophytic, epiphytic, and saprobic species [57]. No data about lipid composition for this family of fungi are available in the literature yet. In the present study, one strain belonging to the Melanommataceae family (UFRO22.418) was assessed for lipid production. According to the obtained data, the Lewis was the best method in FAME recovery (63.7%), followed by the Bligh and Dyer (55.30%) and Folch methods (37.86%).

To date, no information regarding methods for the extraction of lipid content from *Cylindrobasidium eucalypti* is available in the literature. According to the data obtained herein, the Lewis method was the most efficient in recovering FAMEs from *C. eucalypti* UFRO22.226, with a yield of 79.8%, followed by the Bligh and Dyer (28.28%) and Folch (19.86%) methods. Similarly, few studies are available concerning the lipid composition of *Pseudogymnoascus pannorum*. In the present study, the lipid compositions *P. pannorum* UFRO22.138, UFRO22.172, UFRO22.250, and UFRO22.358 were assessed. According to the obtained results, the Lewis method was the best for lipid extraction and FAME recovery, with yields varying from 51.00 to 74.00% among the four assessed strains. For these four strains, the best performance in lipid extraction and FAME recovery was followed by the Bligh and Dyer (from 15.50 to 34.42%) and Folch (from 2.63 to 13.16%) methods (Table 4). Overall, to the best of our knowledge, this is the first study on the evaluation of FAME extraction methods for *Cylindrobasidium eucalypti*, *Penicillium miczynskii*, *P. virgatum*, and *Pseudogymnoascus pannorum*.

### 3.4. Analysing Lipid Extraction with Infrared Spectroscopy

**Figure 2:**
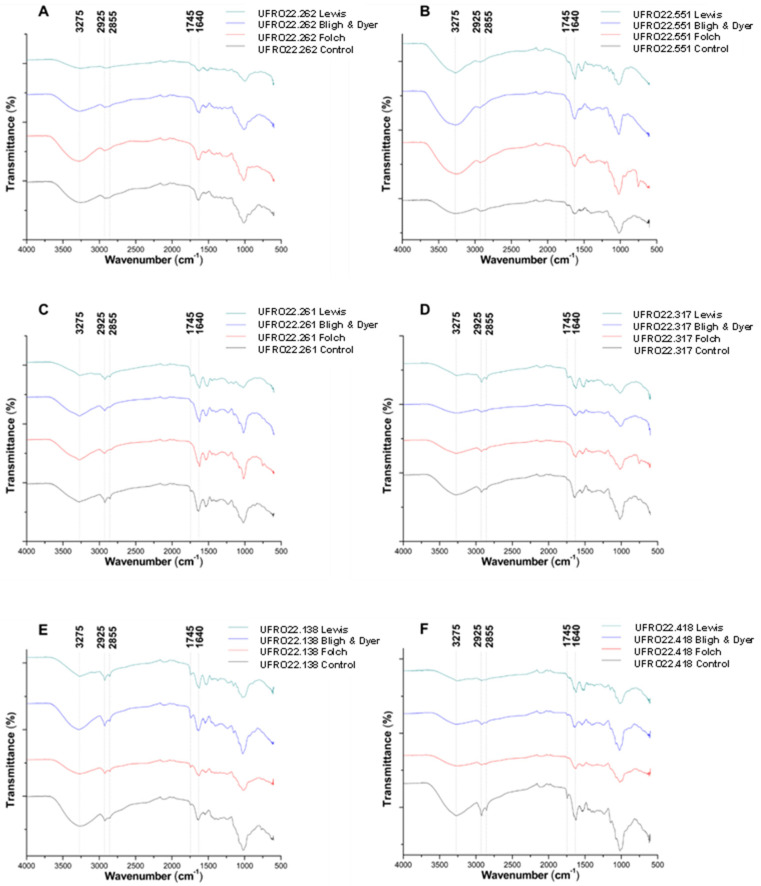
Infrared spectra for fungal biomass. (**A**) *Botrytis cinerea* (UFRO22.262); (**B**) *C. herbarum* (UFRO22.551); (**C**) *M. truficola* (UFRO22.261); (**D**) *M. globulifera* (UFRO22.317); (**E**) *P. pannorum* (UFRO22.138); (**F**) Melanommataceae family (UFRO22.418) before (black line) and after extraction with the Lewis (green line), Bligh and Dyer (blue line), and Folch (red line) methods. The control (biomass before extraction) is presented for each strain.

In order to determine the efficiency of each method in extracting lipids, fungal biomass was subjected to infrared spectroscopy analysis before and after lipid extraction. According to the data obtained in the present study, the infrared spectra of biomass after extraction showed a reduced intensity of bands of amides (3275 and 1640 cm^−1^) and esters (2925, 2855, and 1745 cm^−1^) (Figure 2). These data are in agreement with previous studies developed using infrared spectroscopy analysis to monitor lipid extraction from filamentous fungi [8,9,70]. According to previous studies, in addition to the wavenumbers mentioned above, the lipid content in fungal biomass can generate characteristic bands in the infrared spectrum. These bands are related to C–H stretching vibrations, such as =C–H stretching at 3010 cm^−1^ and C–H stretching in –CH3 and CH2 at 2855, 2925, and 2954 cm^−1^. Other bands, such as C–H bending in CH2 groups (1460 cm^−1^), C–O stretching in C–O–C groups (1070–1250 cm^−1^), and C–H rocking in CH2 groups (720 cm^−1^), can be observed for esters [8,9,70].

In addition to lipid molecules, the fungal cellular content includes other compounds that absorb infrared energy in the above-mentioned wavenumber range. After lipid extraction, the presence of bands in this wavenumber range can be attributed to other compounds, such as proteins and carbohydrates [70]. In the present study, after lipid extraction with the Lewis method, 9 out of 18 (50%) strains (*B. cinerea* UFRO22.262, *C. varians* UFRO22.53, Melanommataceae family UFRO22.418, *M. trufocola* UFRO22.261, *M. globulifera* UFRO22.317, *P. pannorum* UFRO22.138 and UFRO22.250, and *P. virgatum* UFRO22.251 and *P. pannorum* UFRO22.358) presented spectra with decreasing in intensity for bands related to N–H stretching (3275 cm^−1^) and C=O stretching in amides (1640 cm^−1^) (Figure 2). Similar results were observed for 6 out of 18 (c.a. 33%) strains using the Bligh and Dyer method (Melanommataceae family UFRO22.418, *M. globulifera* UFRO22.317, *P. pannorum* UFRO22.172, *P. pannorum* UFRO22.250, *P. virgatum* UFRO22.251, and *P. pannorum* UFRO22.358) (Figures S9–S26).

The spectra obtained for biomass of *P. pannorum* (UFRO22.172) extracted with the Bligh and Dyer method and for biomasses of *M. gamsii* UFRO22.40 and *M. antartica* UFRO22.73 extracted with the Folch method showed the greatest band reductions (Figures S15, S16, and S22, respectively). According to Langseter et al. [70] a decrease in bands related to N–H stretching (3275 cm^−1^) and C=O stretching in amides (1640 cm^−1^) and C–O stretching in esters (1200–1000 cm^−1^) may be due to the co-extraction of other cellular compounds, such as proteins and polysaccharides, which are key components of the fungal cell wall [71]. The authors stated that bands at 3275 cm^−1^ and 1200–1000 cm^−1^ are due to the vibration of N–H, C–O, and C–O–C bonds in compounds such as chitin, chitosan, glucans, and glucuronans from the fungal cell wall. Bands at 875 and 1260 cm^−1^ are due to P–O and P=O stretching, which could be related to the presence of polyphosphates in the cell walls.

Overall, the results obtained herein are in agreement with previous reports in the literature [8,70,71]. In the present study, infrared spectroscopy was an efficient technique for monitoring lipid extraction. The infrared spectroscopy technology used for the analysis does not require prior sample preparation and led to a fast and simple procedure in monitoring lipid extraction from fungal cells.

### 3.5. Fatty Acid Identification and Quantification

**Figure 3:**
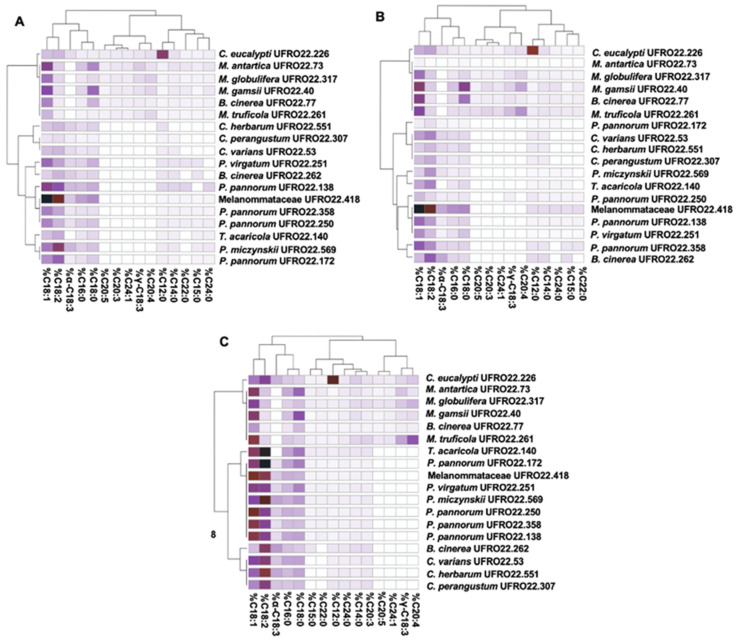
Heat maps of fatty acids from Antarctic fungi according to the performance obtained with the Bligh and Dyer (**A**), Folch (**B**), and Lewis (**C**) methods. The left dendrograms group Antarctic fungi according to similarities of their fatty acid profiles, while the top dendrograms group fatty acids according to their yields. The colour gradient in each heat map varies from darkest (high yield) to lightest (trace yield).

According to the data obtained via chemical characterisation by GC-MS, 17 different FAs were obtained from the assessed fungal strains. Sixteen of them showed long carbon chains (C14–24), with only one FA being exhibiting a medium chain (lauric acid) (Table 5). Among them, one unsaturated FA (UFA), two polyunsaturated FAs (PUFAs), and two saturated FAs (SFAs) were obtained and represented the most abundant lipid molecules in all assessed fungal strains (Figure 3; Tables S2–S5).

These results represent the first report on the FA profiles of different genera and species of filamentous fungi from Fildes Bay, Antarctica. The obtained data are in agreement with data previously reported in the literature [72,73] and offer novel biotechnological perspectives on the application of psychrotolerant fungi. The most common and abundant FAs in Ascomycetes, Basidiomycetes, and Zygomycetes, including oleic, linoleic, palmitic, stearic, and linolenic acids, make up 70–95% of the total FA content [72,73]. Among the fungi assessed in the present study, SFAs (e.g., lauric acid, myristic acid, pentadecanoic acid, behenic acid, and lignoceric acid), UFAs (e.g., nervonic acid), and PUFAs (γ-linolenic acid, α-linolenic acid, dihomo-γ-linolenic acid, arachidonic acid, and 5,8,11,14,17-icosapentaenoic acid) were detected predominantly in lower concentrations or as a trace (Figure 3; Tables S2–S5).

*Botrytis cinerea* has been found to be among the most relevant phytopathogen fungal species, affecting numerous plant hosts, including many important crops, such as carrots, cucumber, tomato, raspberries, and strawberries [74,75]. Despite this, few studies have been focused on the FA profile of *B. cinerea* and its biotechnological potential. The main fatty acids reported for *B. cinerea* are palmitic and linolenic acids (c.a. 23 and 55%, respectively) [59].

In the present study, the FA profiles of the two assessed *B. cinerea* strains (UFRO22.77 and UFRO22.262) differed slightly (Figure 3). For *B. cinerea* UFRO22.262, oleic and linoleic acids were the most prevalent FAs (c.a. 4 and 18%, respectively) (Table S5). Meanwhile, for *B. cinerea* UFRO22.77, stearic and oleic acids were the most prevalent (c.a. 4 and 7%, respectively) (Table S5). In addition, α-linolenic acid was exclusively produced by *B. cinerea* UFRO22.262. The differences in FA profiles between the two *B. cinerea* strains suggests potential differences in metabolic expression in each fungal strain. These differences in metabolic expression are due to the defensive response to environmental conditions of abiotic and/or biotic origin [76].

The *Cladosporium* genus comprises saprobic species (e.g., *C. perangustum*), endophytes (e.g., *C. varians*), plant pathogens, and human allergenic fungal species (e.g., *C. herbarum*) [63,77]. Little information about the FA profiles of *Cladosporium* is available in the literature [78,79,80]. *Cladosporium sphaerospermum*, a widespread mould type, presents an FA profile mainly composed of oleic (c.a. 50%) and palmitic (c.a. 27%) acids [78,79,80].

The *Cladosporium* strains assessed in the present study (*C. herbarum* UFRO22.307, *C. perangustum* UFRO22.53, and *C. varians* UFRO22.551) exhibit different FA compositions among strains, including linoleic (c.a. 20%) and oleic (c.a. 13%) acids (Table S5). Considering the psychrotolerant characteristics of the fungal strains assessed herein, the obtained data suggest that environmental conditions may have an impact on the patterns of the fatty acid profile.

Some species of the *Cylindrobasidium* genus have important biotechnological applications as biocontrol agents and enzyme producers [81]. The present study is the first report on the characterisation of *C. eucalypti* regarding its FA profile. Lauric acid was obtained with a concentration of c.a. 30% for *C. eucalypti* UFRO22.226. This is an important find, as lauric acid has antimicrobial potential. Previous reports have shown a minimal inhibitory concentration of 0.04–0.2 mg/mL of lauric acid against pathogens such as *Aeromonas hydrophila*, *Saprolegnia parasitica*, and *Ichthyophthirius multifiliis* and 0.312–0.625 mg/mL against *Clostridium difficile* [82,83].

The *Mortierella* genus has been widely studied as a lipid producer [84]. Among lipids reported for the *Mortierella* genus, arachidonic acid is a PUFA that has been found in high concentrations in *Mortierella* species (c.a. 40%) [10,85]. PUFAs such as arachidonic acid were obtained from the *M. antartica* UFRO22.73, *M. gamsii* UFRO22.40, *M. globulifera* UFRO22.317, and *M. turficola* UFRO22.261 strains. Among them, *M. turficola* UFRO22.261 produced the highest amount of arachidonic and dihomo-γ-linolenic acids, with c.a. 11 and 3%, respectively. Both arachidonic and dihomo-γ-linolenic acids were found in similar concentrations as those reported in the literature (c.a. 5 and 8%) [9]. Despite this, the data available in the literature for the *Mortierella* genus are mainly focused on just a few model species, such as *M. alpina* [9].

The Trichocomaceae family includes different genera with great potential for FA production, such as *Penicillium* [10,11]. Bardhan et al. [86] assessed a *P. citrinum* strain as potential feedstock for biodiesel production. According to the authors, the observed FA profile was predominantly composed of palmitic (20.25%), oleic (30.09%), and linoleic acids (33.14%). Based on the data obtained herein, *P. miczynskii* UFRO22.569 and *P. virgatum* UFRO22.251 showed FA profiles with similar amounts of oleic (c.a. 13 and 15%, respectively), linoleic (c.a. 29 and 14%, respectively), and palmitic (c.a. 5% for both strains) acids. Despite previous studies on FA profiles of *Penicillium* species, the present study is the first report about the FA compositions of *P. miczynskii* and *P. virgatum* [10,11,86,87,88].

*Pseudogymnoascus pannorum* (formerly *Geomyces pannorum*) is a psychrotolerant fungus widely distributed in cold regions of the Earth [89]. Until now, the available studies have compared the behaviour of *P. pannorum* strains from the polar regions (e.g., the Arctic and the Antarctic) by evaluating the influence of temperature on the FA profile [89,90,91,92,93]. The most commonly reported FAs include oleic, linoleic, palmitic, and stearic acids [89,90,91,92,93]. In addition, Artic *Pseudogymnoascus* strains produce a higher concentration of linolenic acid (c.a. 24%) than Antarctic *Pseudogymnoascus* strains (c.a. 7%). Overall, at low temperatures (c.a. 10 °C), *P. pannorum* strains tend to increase the concentration of oleic acid (c.a. 31–71%) and decrease the concentration of palmitic and stearic acid (c.a. 18–9% and 1–0.5%, respectively) [89,90,91,92,93].

The FA profiles for the *P. pannorum* strains studied herein (UFRO22.172, UFRO22.138, UFRO22.250, and UFRO22.358) are in agreement with those reported in the literature. Both oleic and linoleic acids were found in higher amounts than palmitic and stearic acids (Tables S3–S5). Furthermore, α-linolenic acid was found at low concentrations (c.a. 5%). Therefore, the results obtained here suggest that *Pseudogymnoascus* strains have a more stable metabolism than other fungi assessed in the present study regarding their FA profiles.

*Talaromyces* belongs to the same family as the *Penicillium* genus. When compared with *Penicillium* strains, *T. acaricola* UFRO22.140 presented a different FA profile (Figure 3). To date, there is a limited number of studies focused on *Talaromyces* genera [94]. *Talaromyces thermophilus* has previously been reported to produce oleic and linoleic acids (c.a. 50 and 20%, respectively). The *Talaromyces acaricola* UFRO22.140 strain assessed herein produced oleic and linoleic acids in concentrations of c.a. 19 and 34%, respectively, roughly in agreement with data obtained for *T. thermophilus* by Wright et al. [94]. In addition, *T. acaricola* produced α-Linolenic acid as a trace. Despite this, *T. acaricola* may be a potential source of linoleic acid, an FA in the human diet that is essential for cardiovascular health [95,96].

In the present study, a strain belonging to the Melanomataceae family (UFRO22.418) was assessed for its FA profile. Most species of the Melanommataceae family, such as some species of the *Alpinaria* and *Muriformistrickeria* genera, are saprobic or hyperparasites. These fungi are found on twigs or bark of different woody species in terrestrial, marine, or freshwater habitats. They are widespread in temperate and subtropical regions and can also be found in regions with extreme environments (e.g., Antarctica) [48]. No records about bioactive molecules, such as FAs, are available in the literature on the Melanommataceae family. Consequently, this is the first report about the FA profile of the Melanommataceae family. The lipid profile of the Melanommataceae family (UFRO22.418) is predominantly composed of oleic acid and linoleic acids (c.a. 26 and 20%, respectively), followed by palmitic, stearic, and α-linolenic acids (c.a. 5, 5, and 4%, respectively). The present study provides foundational data for further research into the lipid profiles of the Melanommataceae family.

## 4. Conclusions

According to their carbon percentages, the soil samples from Fildes Bay are similar to mineral soils. Despite this, the presence or absence of vegetation in the place of soil sampling influenced the soil pH. Based on the classical macro- and micro-morphological taxonomy and molecular biology using the ITS region, a total of 17 strains were identified at the species level (*Botrytis cinerea*, *Cladosporium herbarum* complex *herbarum*, *C. perangustum* and *C. varians* complex *cladosporioides*, *C. eucalypti*, *Mortierella antartica*, *M. gamsii*, *M. globulifera*, *M. truficola*, *Penicillium miczynskii*, *P. virgatum*, *Pseudogymnoascus pannorum*, *P. pannorum*, *P. pannorum*, *P. pannorum*, and *Talaromyces acaricola* sect. *Islandici*), and 1 strain was classified at the family level (Melanommataceae). This suggests the need to use other molecular markers for identification at the genus and species levels of certain groups of fungi, such as those belonging to the Melanommataceae family.

In addition, this study provides the first analysis of FA extraction methods and FA identification from *C. eucalypti*, *Penicillium miczynskii*, *P. virgatum*, and *Pseudogymnoascus pannorum*. The data presented herein show that the Lewis method was the best in recovering FAMEs from fungal biomass. Although the Bligh and Dyer and Folch methods were useful for FA extraction, both methods were less efficient in recovering FAME. The assessed Antarctic filamentous fungi produced a pool of FA, emphasising their biotechnological potential as oil producers.

Infrared spectroscopy was a useful method for monitoring the lipid extraction procedures, while gas chromatography led to a comprehensive characterisation and was able to quantify the obtained FAMEs. A total of 17 FAs were identified, and the FA profile varied among fungal species and strains. The FA profiles among the different strains of *B. cinerea* and *P. pannorum* analysed in the present study differed considerably. In addition to the extraction method used, the abundance of FA extracted also depended on the intrinsic capacity of each fungal species and strain. For the three assessed methods, UFA was the most abundant class of obtained lipid, among which oleic and linoleic acids were the most predominant in all fungal strains, followed by SFAs such as stearic and palmitic acids.

In the present study, in addition to fungal species well known for their oil-producing potential (e.g., *Mortierella* spp.), other fungal species that have been minimally explored for lipid production in the past, such as *C. eucalypti*, *P. miczynskii*, *P. virgatum*, and *Pseudogymnoascus pannorum*, were observed to be promising alternative FA sources.

## Figures and Tables

**Table 1:** Results of chemical analysis of Antarctic soil samples.

Sample	Na	K	Ca	Mg	P	pH	C/N	%Nt	%OC
	mg/kg								
S1	74.4	149.3	476.4	476.4	71.4	6.5	43.7	0.1	3.0
S3	256.2	147.8	2067.5	2067.5	125.8	6.4	20.5	0.4	7.8
S4	254.5	138.8	757.6	757.6	274.2	6.6	26.7	0.4	9.7
S5	180.6	136.8	4363.4	4363.4	370.7	6.2	1.6	2.3	3.7
S6	164.2	132.6	457.1	32.8	81.2	7.5	5.3	0.5	2.4
S7	194.3	153.5	974.4	54.9	26.1	7.2	54.3	0.2	11.9
S8	300.0	145.8	1058.3	1058.3	186.7	6.9	6.5	0.4	2.7
S9	454.0	175.5	1152.7	1152.7	62.4	6.7	6.0	1.0	5.8
S10	726.2	265.2	1886.2	155.5	3.6	7.4	25.4	0.2	5.9
S11	188.3	161.0	1354.5	71.0	31.6	7.1	15.6	0.7	11.4

**Table 2:** Antarctic fungal strains isolated and used in the present study.

UFRO Access	Taxonomy
UFRO22.77	*Botrytis cinerea*
UFRO22.262	*Botrytis cinerea*
UFRO22.551	*Cladosporium herbarum* complex *herbarum*
UFRO22.307	*Cladosporium perangustum* complex cladosporioides
UFRO22.53	*Cladosporium varians* complex cladosporioides
UFRO22.226	*Cylindrobasidium eucalypti*
UFRO22.73	*Mortierella antartica*
UFRO22.40	*Mortierella gamsii*
UFRO22.317	*Mortierella globulifera*
UFRO22.261	*Mortierella truficola*
UFRO22.569	*Penicillium miczynskii*
UFRO22.251	*Penicillium virgatum*
UFRO22.138	*Pseudogymnoascus pannorum*
UFRO22.172	*Pseudogymnoascus pannorum*
UFRO22.250	*Pseudogymnoascus pannorum*
UFRO22.358	*Pseudogymnoascus pannorum*
UFRO22.140	*Talaromyces acaricola* sect. *Islandici*
UFRO22.418	Melanommataceae family

**Table 3:** Effectiveness assessment of lipid extraction among methods.

	Bligh and Dyer				Folch				Lewis			
	X	SD		L	X		SD	L	X		SD	L
TLE%	3.83%	±	4.25	b	8.64%	±	8.36	a	7.54%	±	3.86	A
FAMEs	0.89%	±	1.11	c	1.13%	±	1.62	b	4.01%	±	1.86	A

TLE%: total lipid extract; FAMEs: methyl esters of fatty acids; X: average; SD: standard deviation; L: statistical significance letter. Different letters indicate statistical significant difference in the data available in each line (*p* < 0.05).

**Table 4:** Effectiveness assessment of FAME extraction for each fungal strain.

UFRO Accesses	Taxonomy	Bligh and Dyer	Folch	Lewis
UFRO22.77	*Botrytis cinerea*	16.46%	15.87%	20.70%
UFRO22.262	*Botrytis cinerea*	13.92%	13.18%	45.73%
UFRO22.551	*Cladosporium herbarum* complex herbarum	12.29%	6.94%	53.58%
UFRO22.307	*Cladosporium perangustum* complex cladosporioides	5.33%	6.80%	42.73%
UFRO22.53	*Cladosporium varians* complex cladosporioides	11.26%	9.74%	50.06%
UFRO22.226	*Cylindrobasidium eucalypti*	28.28%	19.86%	79.80%
UFRO22.73	*Mortierella antartica*	30.68%	1.25%	51.93%
UFRO22.40	*Mortierella gamsii*	26.65%	23.42	46.76%
UFRO22.317	*Mortierella globulifera*	19.58%	11.30%	45.71%
UFRO22.261	*Mortierella truficola*	11.75%	18.25%	60.34%
UFRO22.569	*Penicillium miczynskii*	28.62%	6.52%	65.85%
UFRO22.251	*Penicillium virgatum*	22.24%	7.90%	51.01%
UFRO22.138	*Pseudogymnoascus pannorum*	34.42%	10.60%	55.19%
UFRO22.172	*Pseudogymnoascus pannorum*	18.09%	2.63%	74.00%
UFRO22.250	*Pseudogymnoascus pannorum*	17.88%	6.41%	59.59%
UFRO22.358	*Pseudogymnoascus pannorum*	15.50%	13.16%	51.00%
UFRO22.140	*Talaromyces acaricola* sect. *Islandici*	11.55%	8.04%	76.23%
UFRO22.418	Melanommataceae family	55.30%	37.86%	63.70%

**Table 5:** GC-MS identification of FAs obtained from Antarctic fungi using the Bligh and Dyer, Folch, and Lewis methods.

Fatty Acid	Chain Length	Retention Time (min)
Lauric acid	C12:0	12.008
Myristic acid	C14:0	12.790
Pentadecanoic acid	C15:0	13.215
Palmitic acid	C16:0	13.710
Palmitoleic acid	C16:1	14.166
Margaric acid	C17:0	14.166
Stearic acid	C18:0	14.861
Oleic acid	C18:1	15.411
Linoleic acid	C18:2	16.262
γ-Linolenic acid	γ-C18:3	17.381
α-Linolenic acid	α-C18:3	17.381
Behenic acid	C22:0	18.201
Dihomo-γ-Linolenic acid	C20:3	19.188
Arachidonic acid	C20:4	20.096
Lignoceric acid	C24:0	21.009
5,8,11,14,17-Eicosapentaenoic acid	C20:5	22.185
Nervonic acid	C24:1	22.379

## Data Availability

The original contributions presented in this study are included in the article/Supplementary Materials. Further inquiries can be directed to the corresponding authors.

## Supplementary Materials

The following supporting information can be downloaded at: https://www.mdpi.com/article/10.3390/microorganisms13030504/s1, Figure S1: Phylogenetic tree of *Botrytis* genera based on ITS sequences; Figure S2: Phylogenetic tree of *Cladosporium* genera based on ITS sequences; Figure S3: Phylogenetic tree of *Cylindrobasidium* genera based on ITS sequences; Figure S4: Phylogenetic tree of *Mortierella* genera based on ITS sequences; Figure S5: Phylogenetic tree of *Penicillium* genera based on ITS sequences; Figure S6: Phylogenetic tree of *Pseudogymnoascus* genera based on ITS sequences; Figure S7: Phylogenetic tree of *Talaromyces* genera based on ITS sequences; Figure S8: Phylogenetic tree of the Melanomataceae family based on ITS sequences; Table S1: Effectiveness of lipid extraction in each fungal strain; Table S2: Average quantification of each fatty acid obtained with the Bligh and Dyer, Folch, and Lewis methods; Table S3: Fatty acid yields quantified in all extracts, obtained by the Bligh and Dyer method; Table S4: Fatty acid yields quantified in all extracts, obtained by the Folch method; Table S5: Fatty acid yields quantified in all extracts, obtained by the Lewis method; Table S6: Antarctic fungal strains isolated and used in the present study; Figure S9: Infrared spectra for fungal biomass; Figure S10: Infrared spectra for fungal biomass; Figure S11: Infrared spectra for fungal biomass; Figure S12: Infrared spectra for fungal biomass; Figure S13: Infrared spectra for fungal biomass; Figure S14: Infrared spectra for fungal biomass in *Cylindrobasidium eucalypti* (UFRO22.226); Figure S15: Infrared spectra for fungal biomass in *Mortierella antartica* (UFRO22.73); Figure S16: Infrared spectra for fungal biomass in *Mortierella gamsii* (UFRO22.40); Figure S17: Infrared spectra for fungal biomass in *Mortierella globulifera* (UFRO22.317); Figure S18: Infrared spectra for fungal biomass in *Mortierella truficola* (UFRO22.261); Figure S19: Infrared spectra for fungal biomass in *Penicillium miczynskii* (UFRO22.569); Figure S20: Infrared spectra for fungal biomass in *Penicillium virgatum* (UFRO22.251); Figure S21: Infrared spectra for fungal biomass in *Pseudogymnoascus pannorum* (UFRO22.138); Figure S22: Infrared spectra for fungal biomass in *Pseudogymnoascus pannorum* (UFRO22.172); Figure S23: Infrared spectra for fungal biomass in *Pseudogymnoascus pannorum* (UFRO22.250); Figure S24: Infrared spectra for fungal biomass in *Pseudogymnoascus pannorum* (UFRO22.358); Figure S25: Infrared spectra for fungal biomass in *Talaromyces acaricola* sect. *Islandici* (UFRO22.140); Figure S26: Infrared spectra for fungal biomass in the Melanommataceae family (UFRO22.418).
