# Supplementary material for: Lipid Production in Cultivable Filamentous Fungi Isolated from Antarctic Soils: A Comprehensive Study
Source: Microorganisms. 2025 Feb 25;13(3):504. doi: 10.3390/microorganisms13030504 (PMC11944995; doi:10.3390/microorganisms13030504)
Supplement: Supplementary file 1 [file microorganisms-13-00504-s001.zip › Supplementary Material_Gallardo et al. 2025.pdf]

Supplementary Materials:

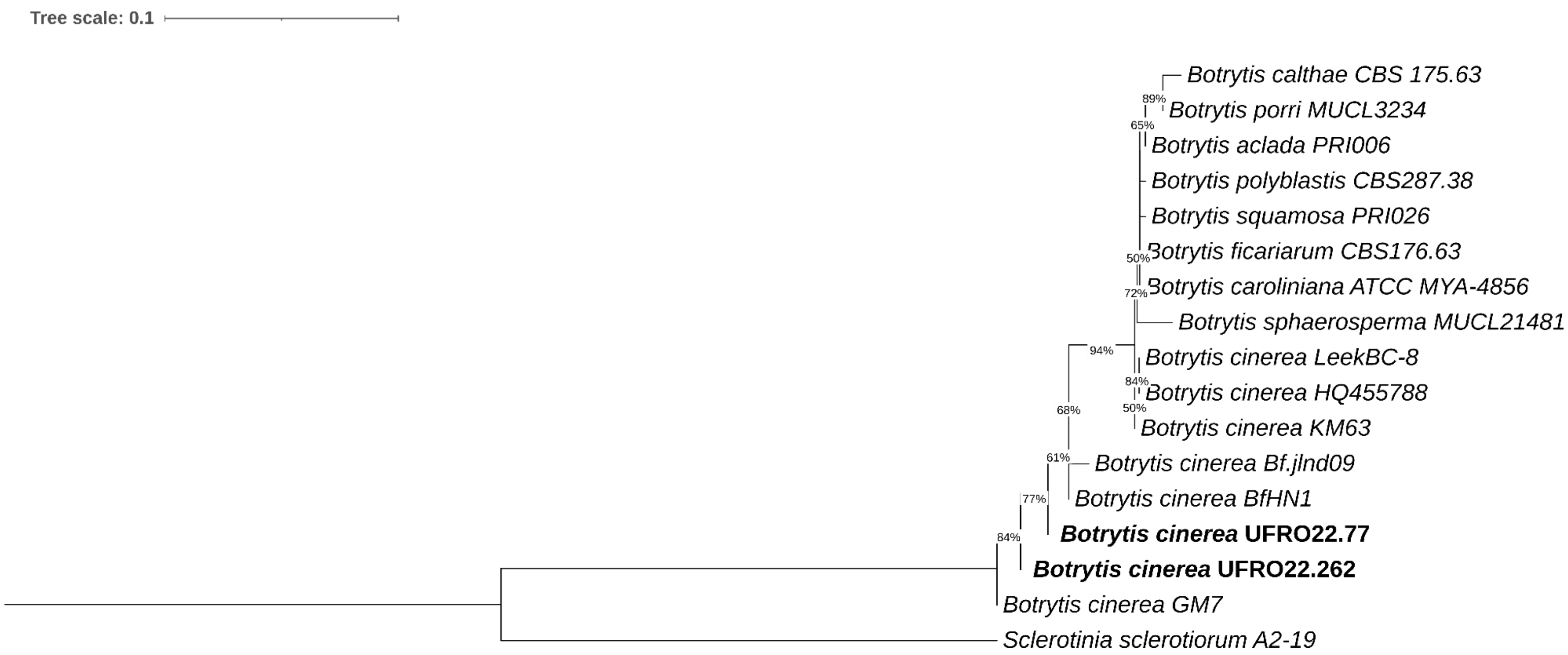

Figure S1. Phylogenetic tree of genera *Botrytis* based on ITS sequences. The method is based on best-scoring maximum likelihood. ITS sequences were obtained in Genebank. Bootstrap support for internal branches is indicated as percentages. The Antarctic strains of this study were highlighted in bold. *Sclerotinia sclerotium* A2-19 was used as an outgroup.

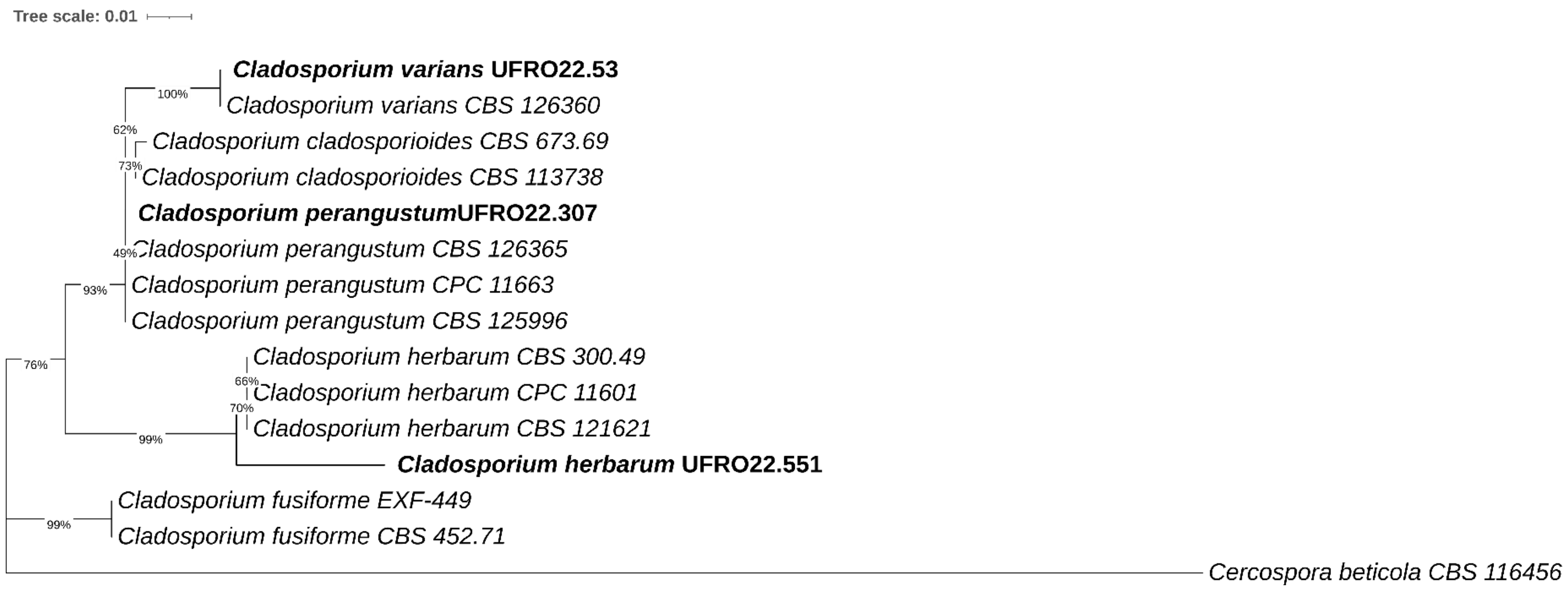

Figure S2. Phylogenetic tree of genera *Cladosporium* based on ITS sequences. The method is based on best-scoring maximum likelihood. ITS sequences were obtained in Genebank. Bootstrap support for internal branches is indicated as percentages. The Antarctic strains of this study were highlighted in bold. *Cercospora beticola* CBS 116456 was used as an outgroup.

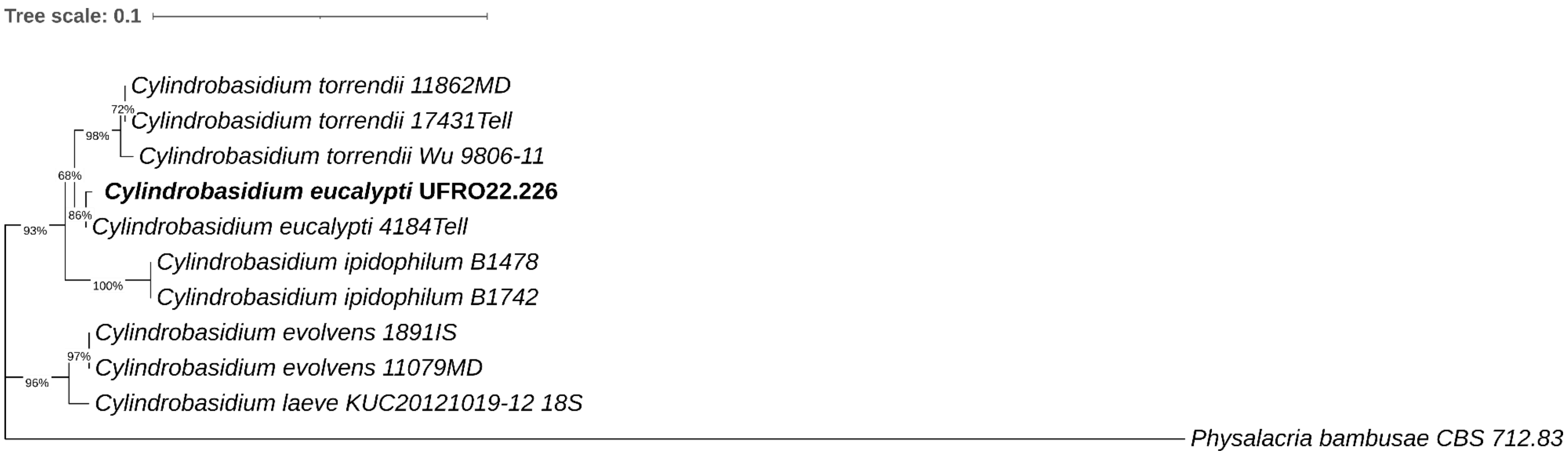

Figure S3. Phylogenetic tree of genera *Cylindrobasidium* based on ITS sequences. The method is based on best-scoring maximum likelihood. ITS sequences were obtained in Genebank. Bootstrap support for internal branches is indicated as percentages. The Antarctic strains of this study was highlighted in bold. *Physalacria bambusae* CBS 712.83 was used as an outgroup.

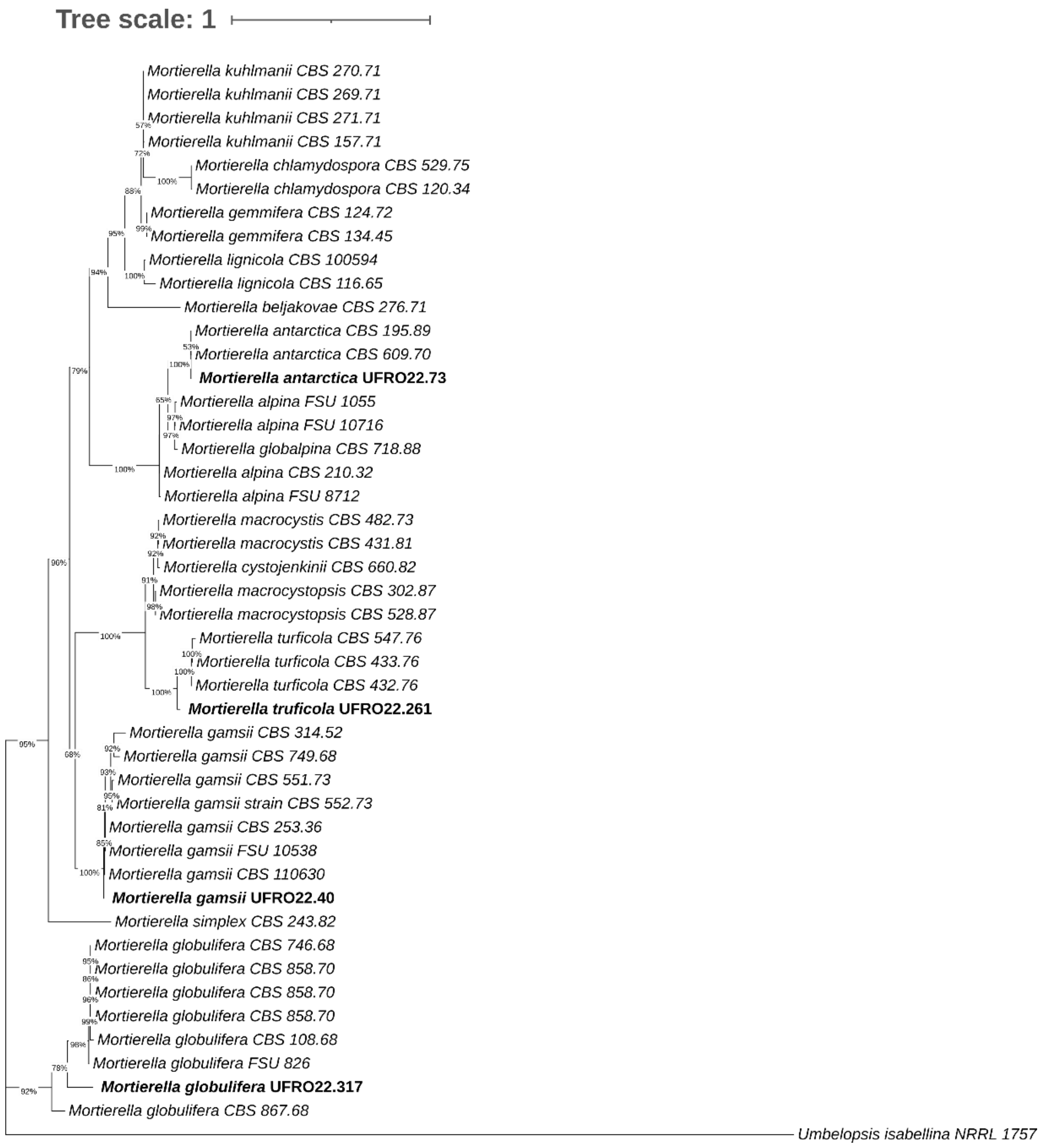

Figure S4. Phylogenetic tree of genera *Mortierella* based on ITS sequences. The method is based on best-scoring maximum likelihood. ITS sequences were obtained in Genebank. Bootstrap support for internal branches is indicated as percentages. The Antarctic strains of this study were highlighted in bold. *Umbelopsis isabellina* NRRL 1757 was used as an outgroup.

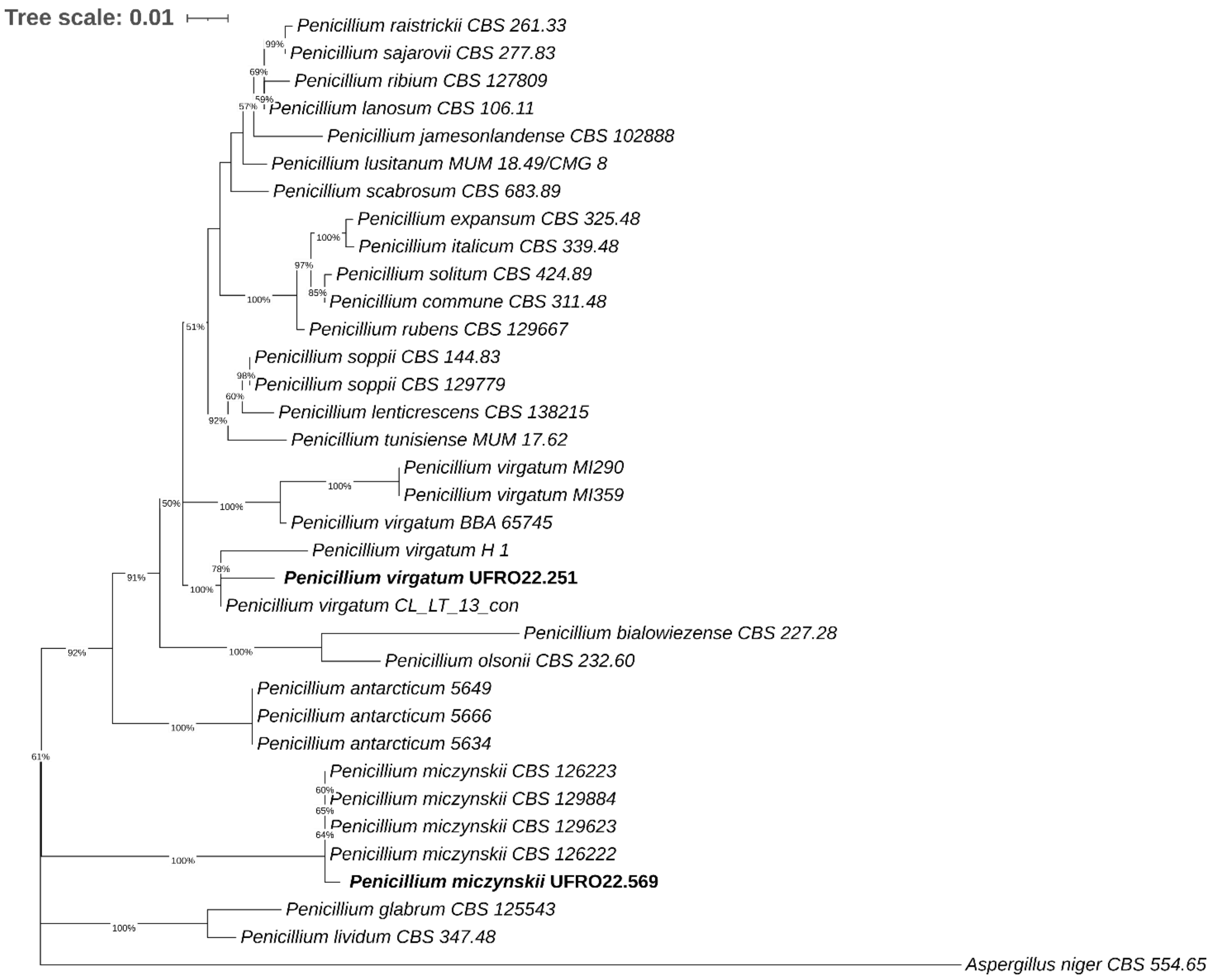

Figure S5. Phylogenetic tree of genera *Penicillium* based on ITS sequences. The method is based on best-scoring maximum likelihood. ITS sequences were obtained in Genebank. Bootstrap support for internal branches is indicated as percentages. The Antarctic strains of this study were highlighted in bold. *Aspergillus niger* CBS 554.65 was used as an outgroup.

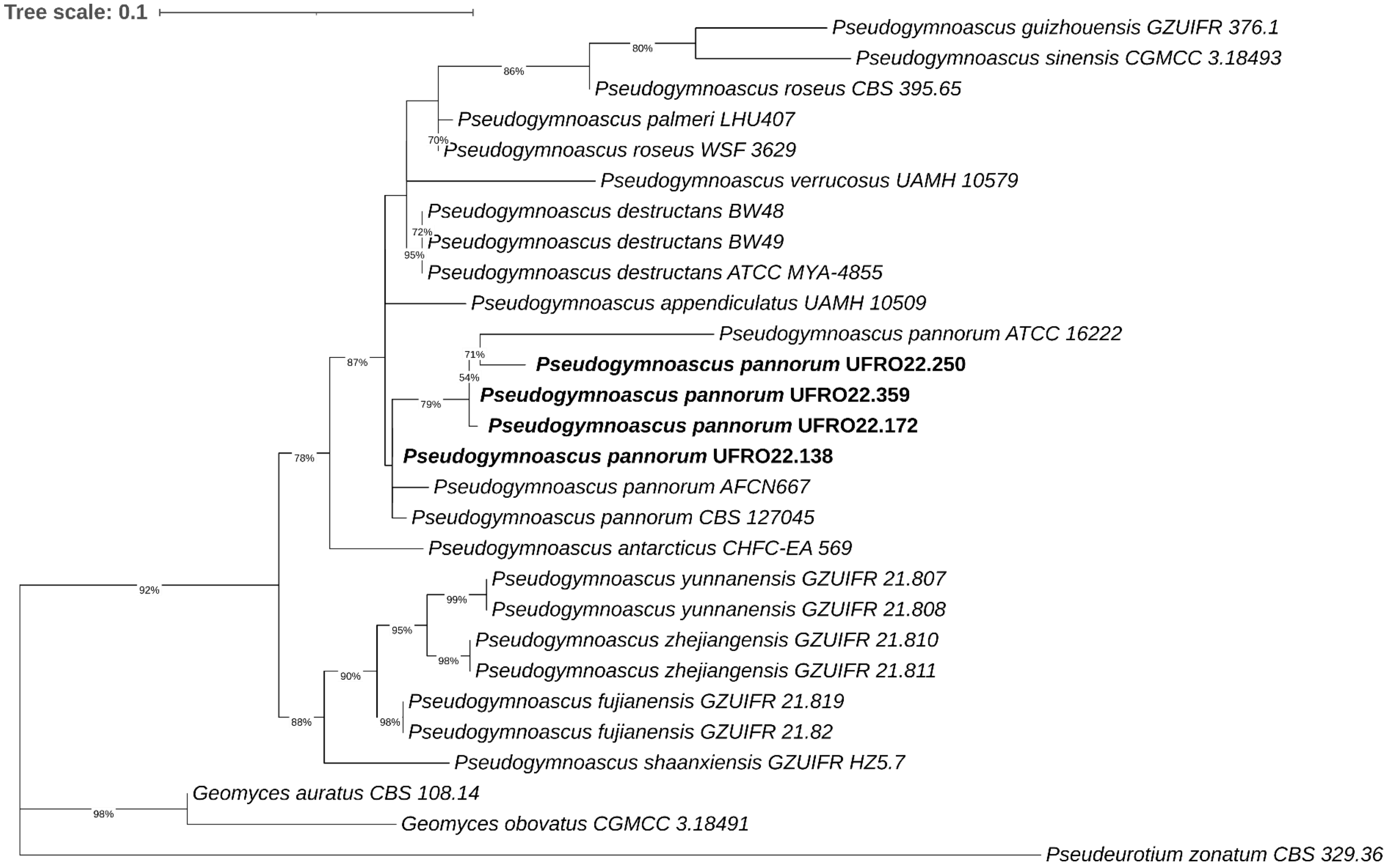

Figure S6. Phylogenetic tree of genera *Pseudogymnoascus* based on ITS sequences. The method is based on best-scoring maximum likelihood. ITS sequences were obtained in Genebank. Bootstrap support for internal branches is indicated as percentages. The Antarctic strains of this study were highlighted in bold. *Pseudoterium zonatum* CBS 329.36 was used as an outgroup.

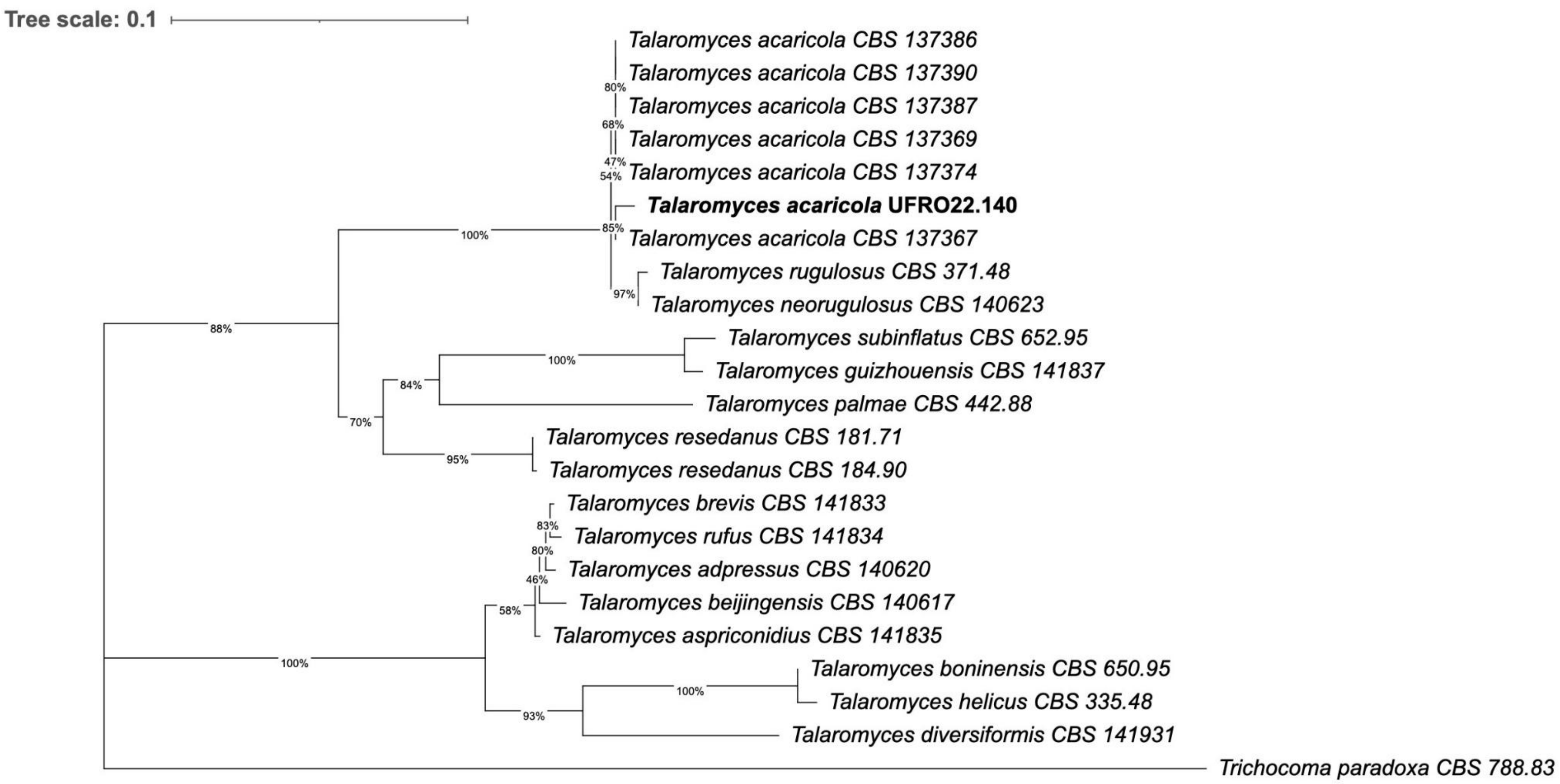

Figure S7. Phylogenetic tree of genera *Talaromyces* based on ITS sequences. The method is based on best-scoring maximum likelihood. ITS sequences were obtained in Genebank. Bootstrap support for internal branches is indicated as percentages. The Antarctic strain of this study was highlighted in bold. *Trichocoma paradoxa* CBS 788.83 T was used as an outgroup.

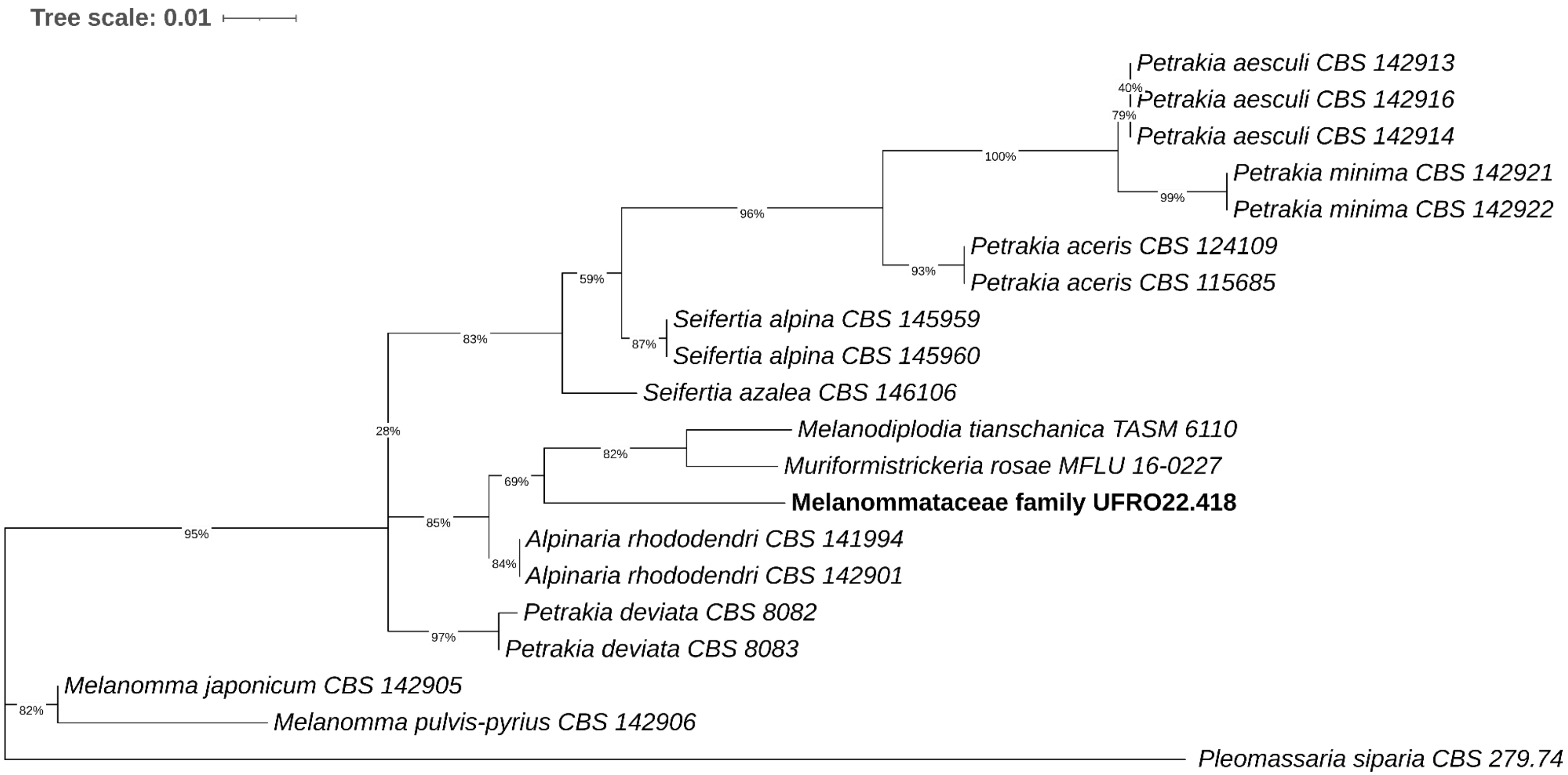

Figure S8. Phylogenetic tree of the Melanomataceae family based on ITS sequences. The method is based on best-scoring maximum likelihood. ITS sequences were obtained in Genebank. Bootstrap support for internal branches is indicated as percentages. The Antarctic strain of this study was highlighted in bold. *Pleomassaria siparia* CBS 279.74 was used as an outgroup.

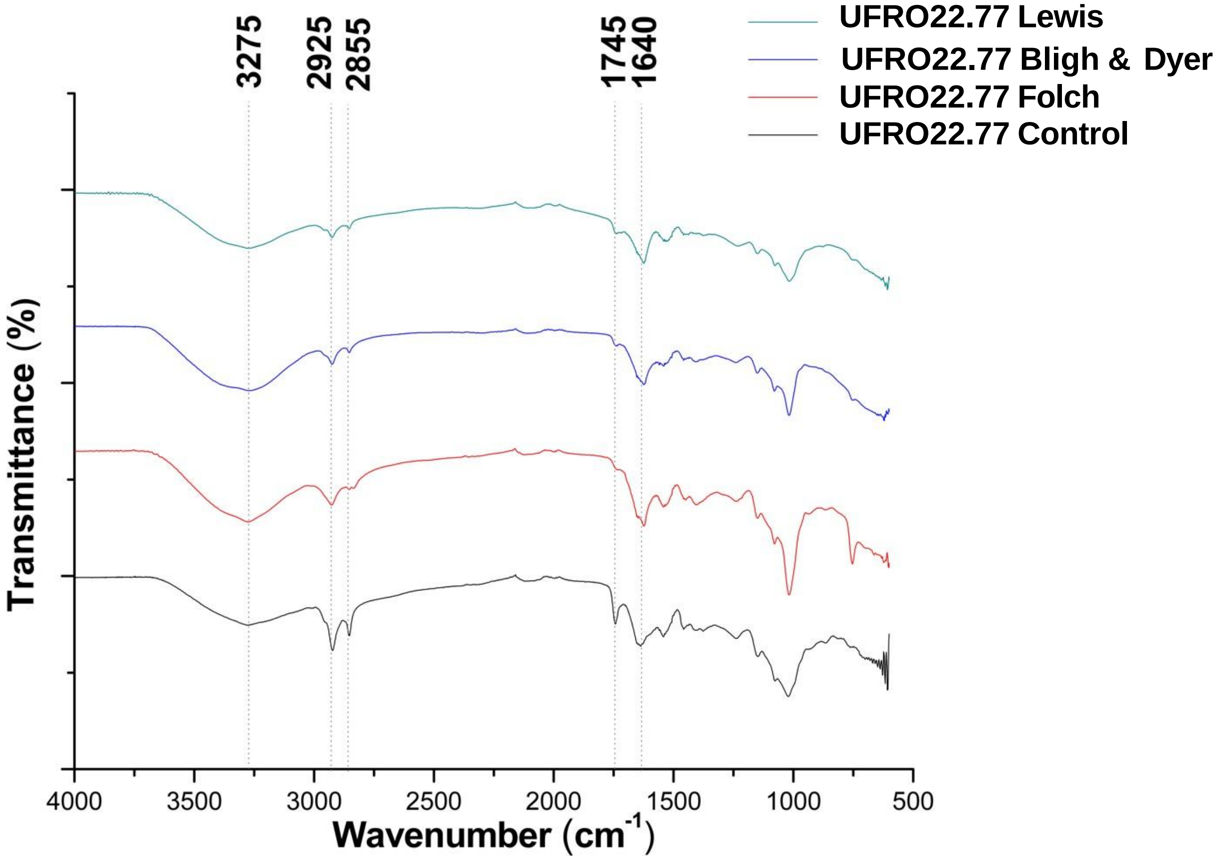

Figure S9. Infrared spectra for fungi biomass. *Botrytis cinerea* (UFRO22.77), before (black line) and after extraction with Lewis (green line), Bligh & Dyer (blue line), Folch (red line) methods. Control is presented for each strain.

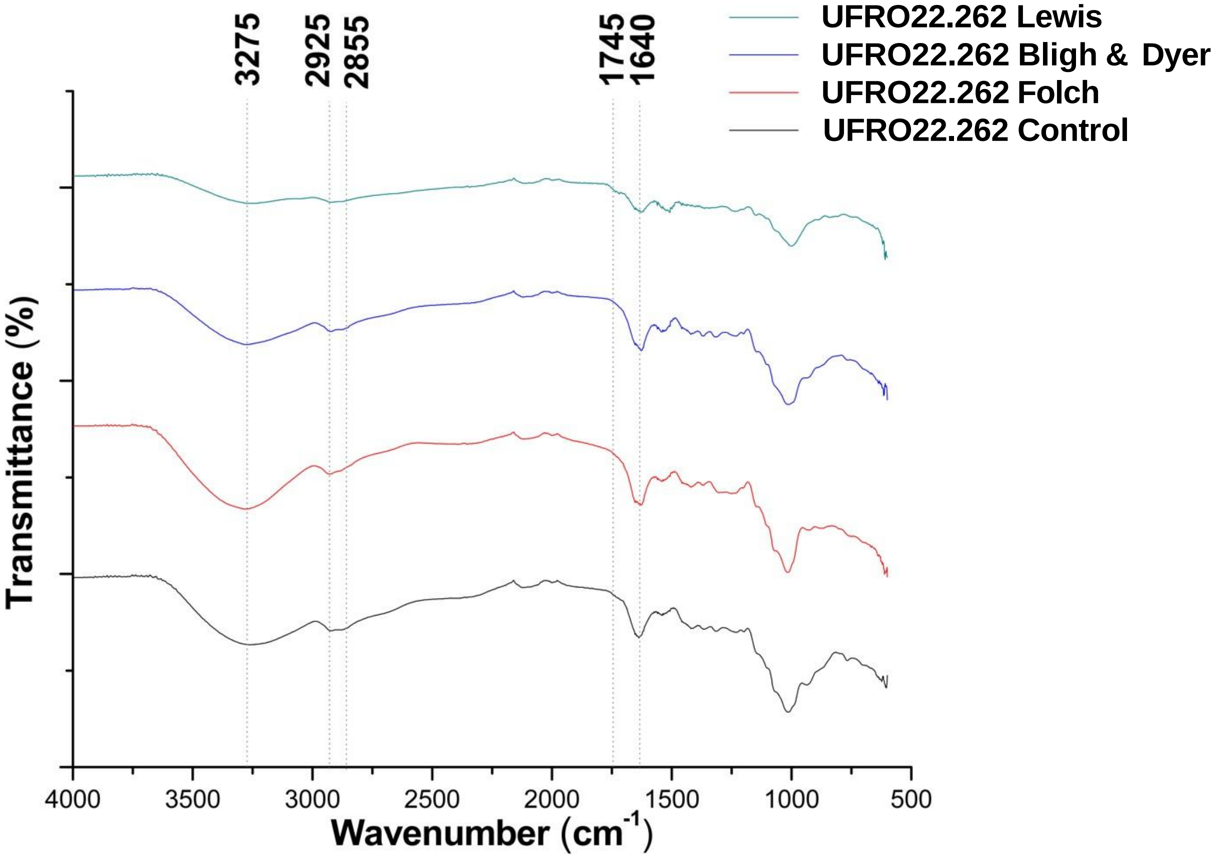

Figure S10. Infrared spectra for fungi biomass. *Botrytis cinerea* (UFRO22.262), before (black line) and after extraction with Lewis (green line), Bligh & Dyer (blue line), Folch (red line) methods. Control is presented for each strain.

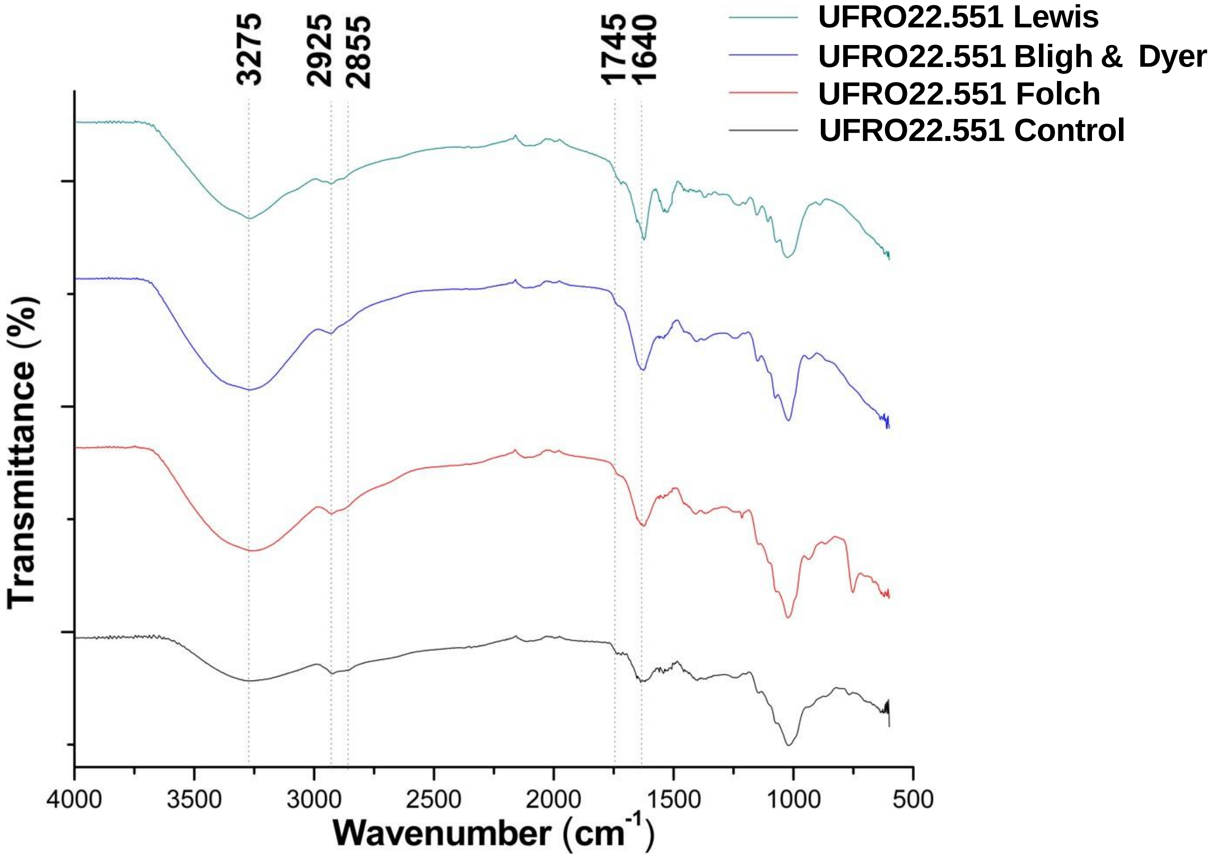

Figure S11. Infrared spectra for fungi biomass. *Cladosporium herbarum* complex *herbarum* (UFRO22.551), before (black line) and after extraction with Lewis (green line), Bligh & Dyer (blue line), Folch (red line) methods. Control is presented for each strain.

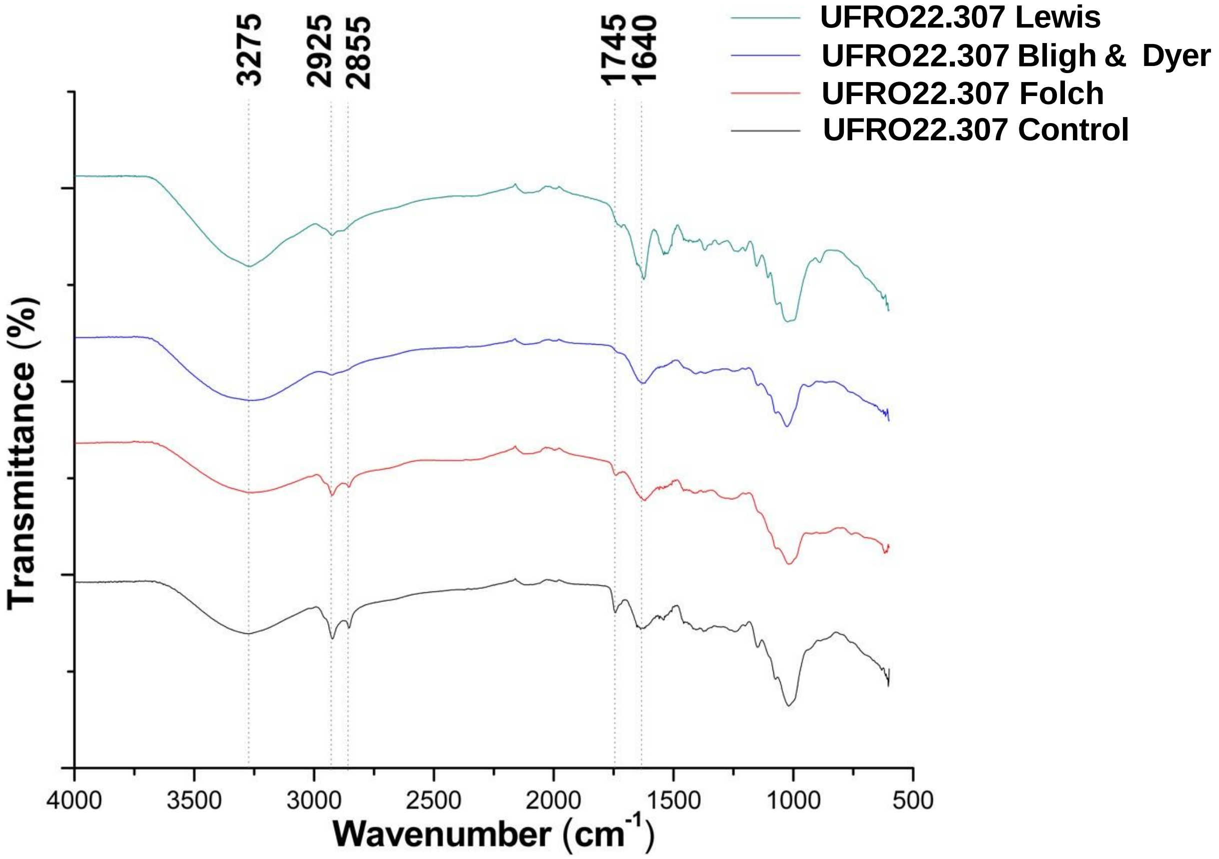

Figure S12. Infrared spectra for fungi biomass. *Cladosporium perangustum* complex *cladosporioides* (UFRO22.307), before (black line) and after extraction with Lewis (green line), Bligh & Dyer (blue line), Folch (red line) methods. Control is presented for each strain.

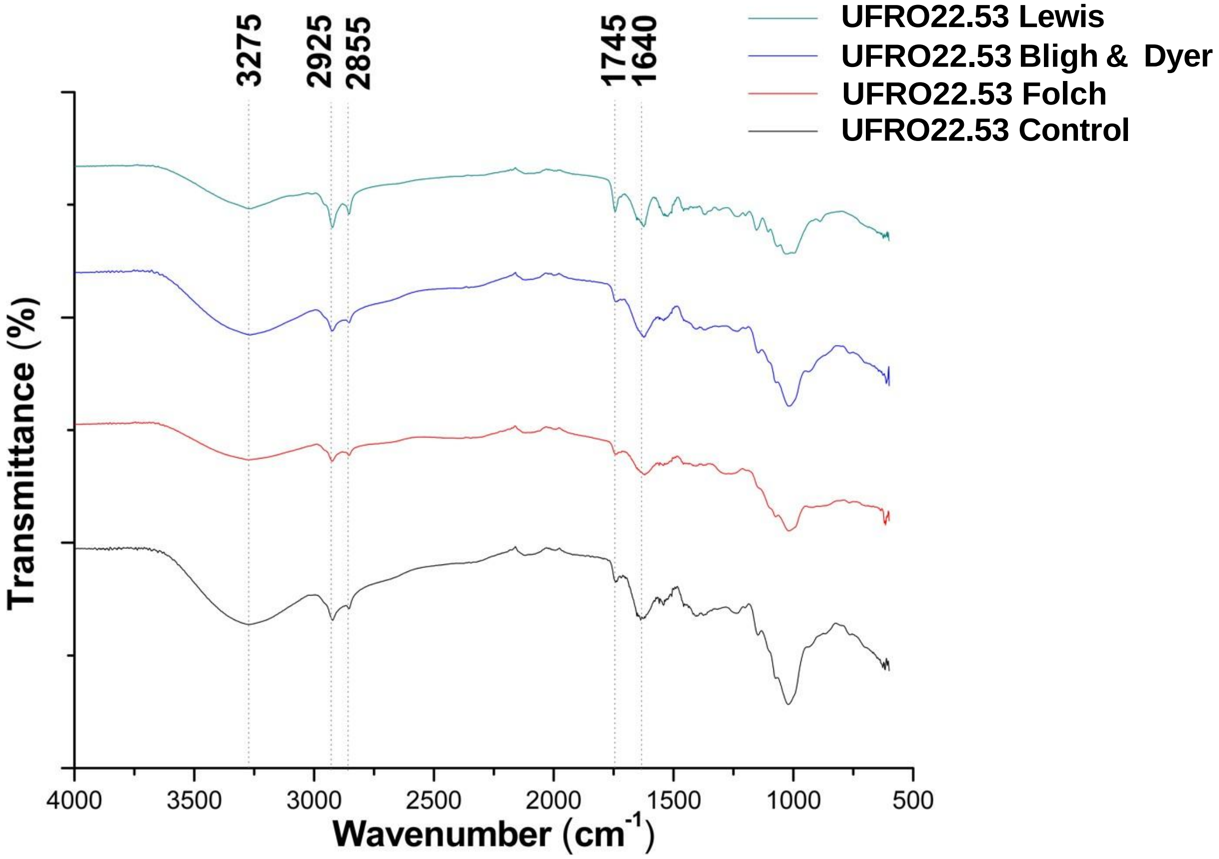

Figure S13. Infrared spectra for fungi biomass. *Cladosporium varians* complex *cladosporioides* (UFRO22.53), before (black line) and after extraction with Lewis (green line), Bligh & Dyer (blue line), Folch (red line) methods. Control is presented for each strain.

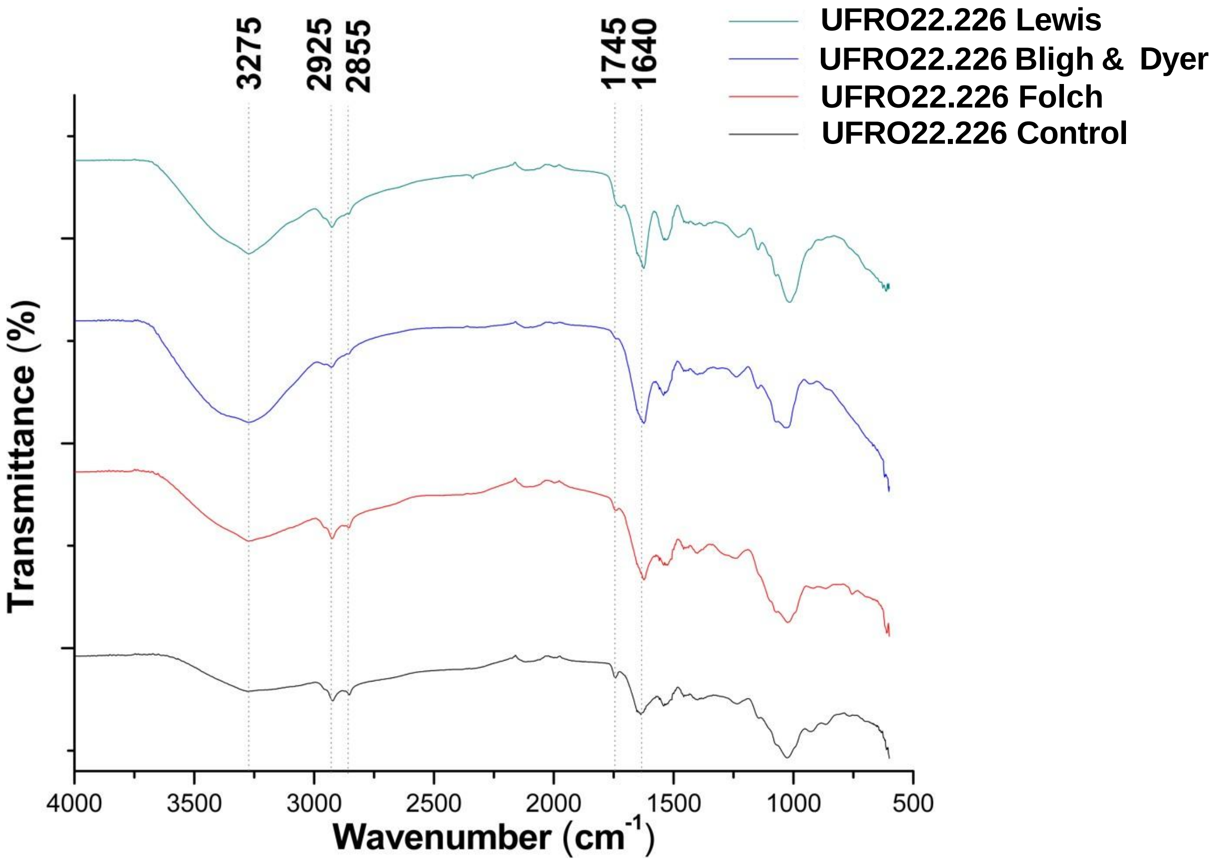

Figure S14. Infrared spectra for fungi biomass *Cylindrobasidium eucalypti* (UFRO22.226), before (black line) and after extraction with Lewis (green line), Bligh & Dyer (blue line), Folch (red line) methods. Control is presented for each strain.

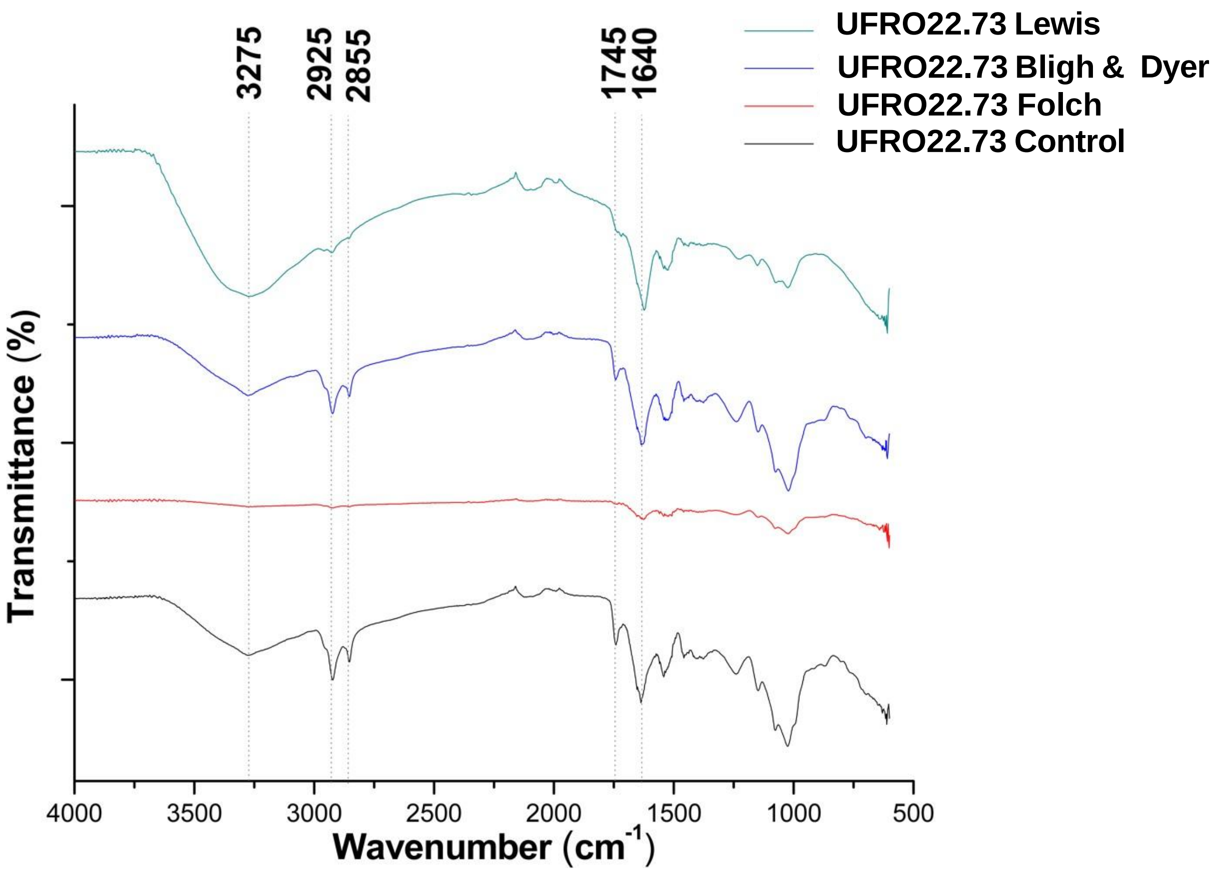

Figure S15. Infrared spectra for fungi biomass *Mortierella antartica* (UFRO22.73), before (black line) and after extraction with Lewis (green line), Bligh & Dyer (blue line), Folch (red line) methods. Control is presented for each strain.

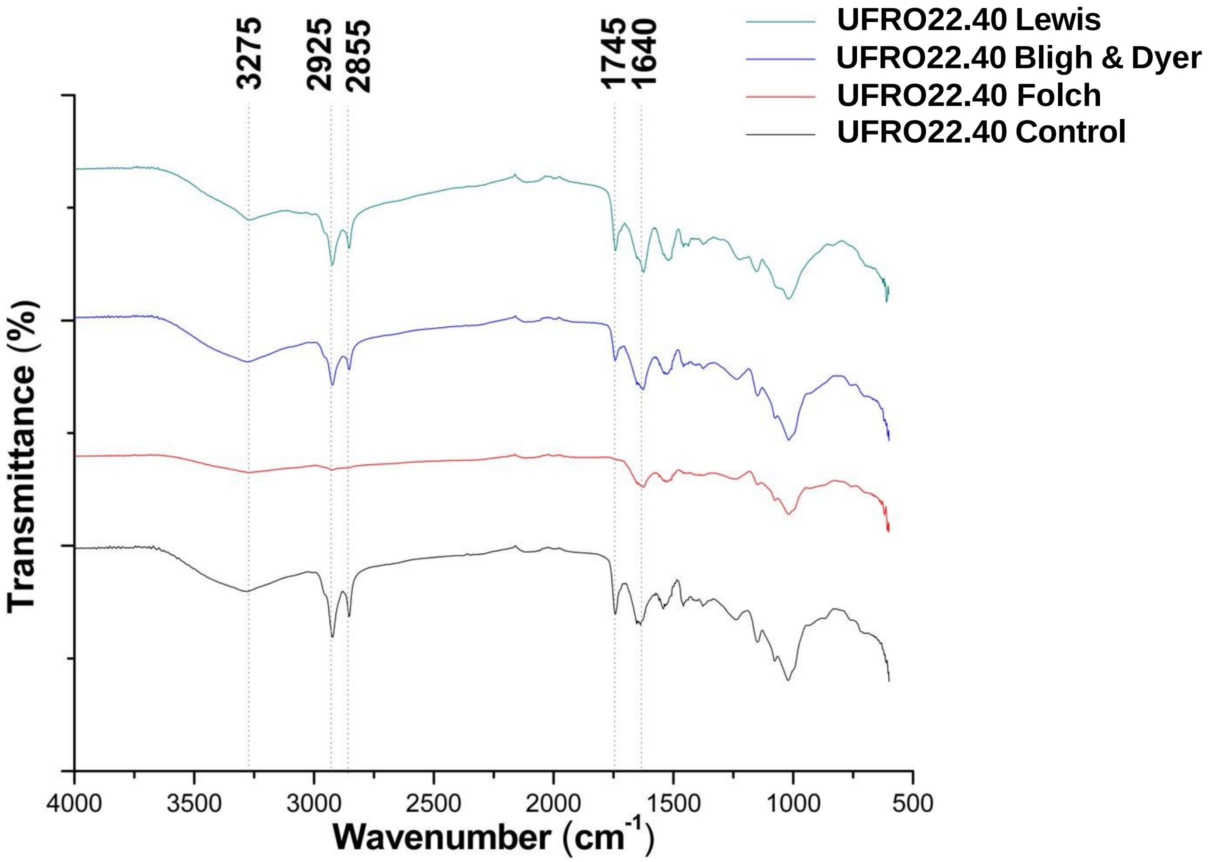

Figure S16. Infrared spectra for fungi biomass *Mortierella gamsii* (UFRO22.40), before (black line) and after extraction with Lewis (green line), Bligh & Dyer (blue line), Folch (red line) methods. Control is presented for each strain.

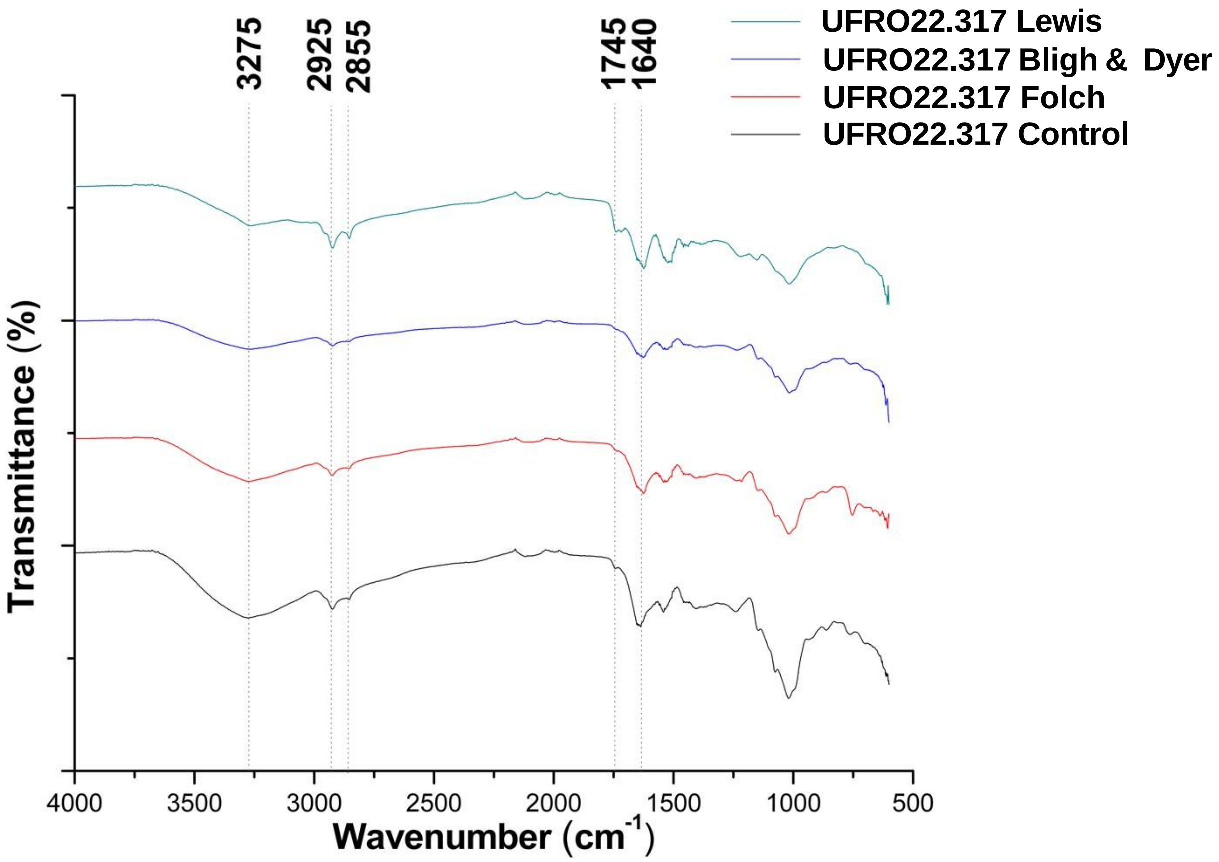

Figure S17. Infrared spectra for fungi biomass *Mortierella globulifera* (UFRO22.317), before (black line) and after extraction with Lewis (green line), Bligh & Dyer (blue line), Folch (red line) methods. Control is presented for each strain.

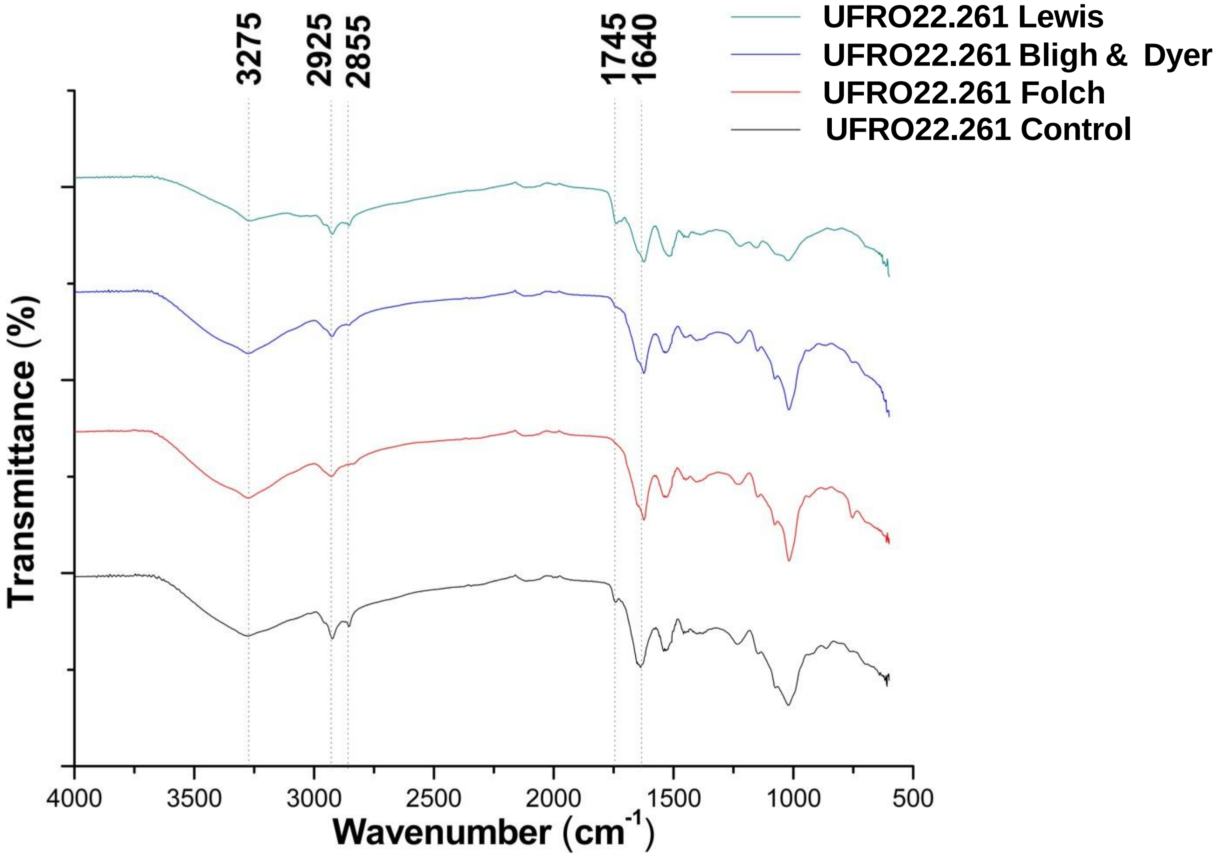

Figure S18. Infrared spectra for fungi biomass *Mortierella truficola* (UFRO22.261), before (black line) and after extraction with Lewis (green line), Bligh & Dyer (blue line), Folch (red line) methods. Control is presented for each strain.

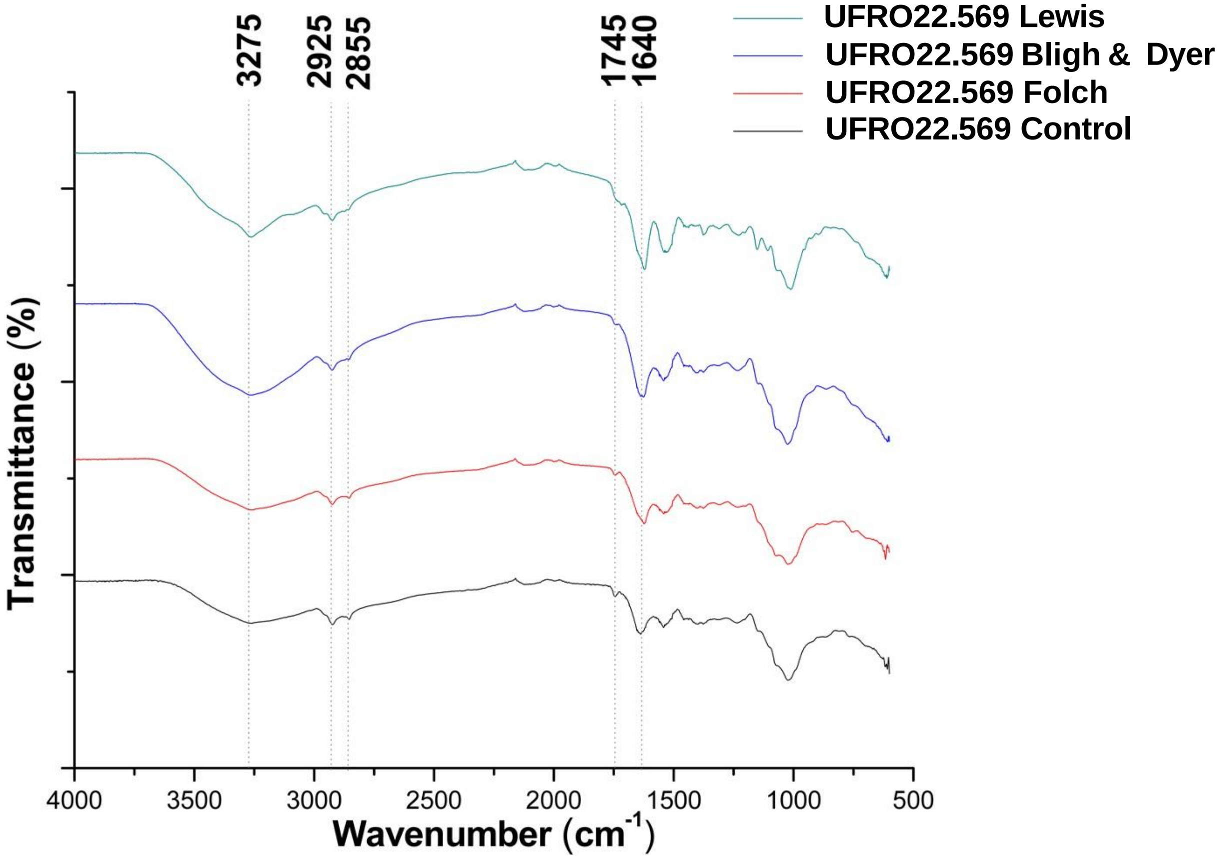

Figure S19. Infrared spectra for fungi biomass *Penicillium miczynskii* (UFRO22.569), before (black line) and after extraction with Lewis (green line), Bligh & Dyer (blue line), Folch (red line) methods. Control is presented for each strain.

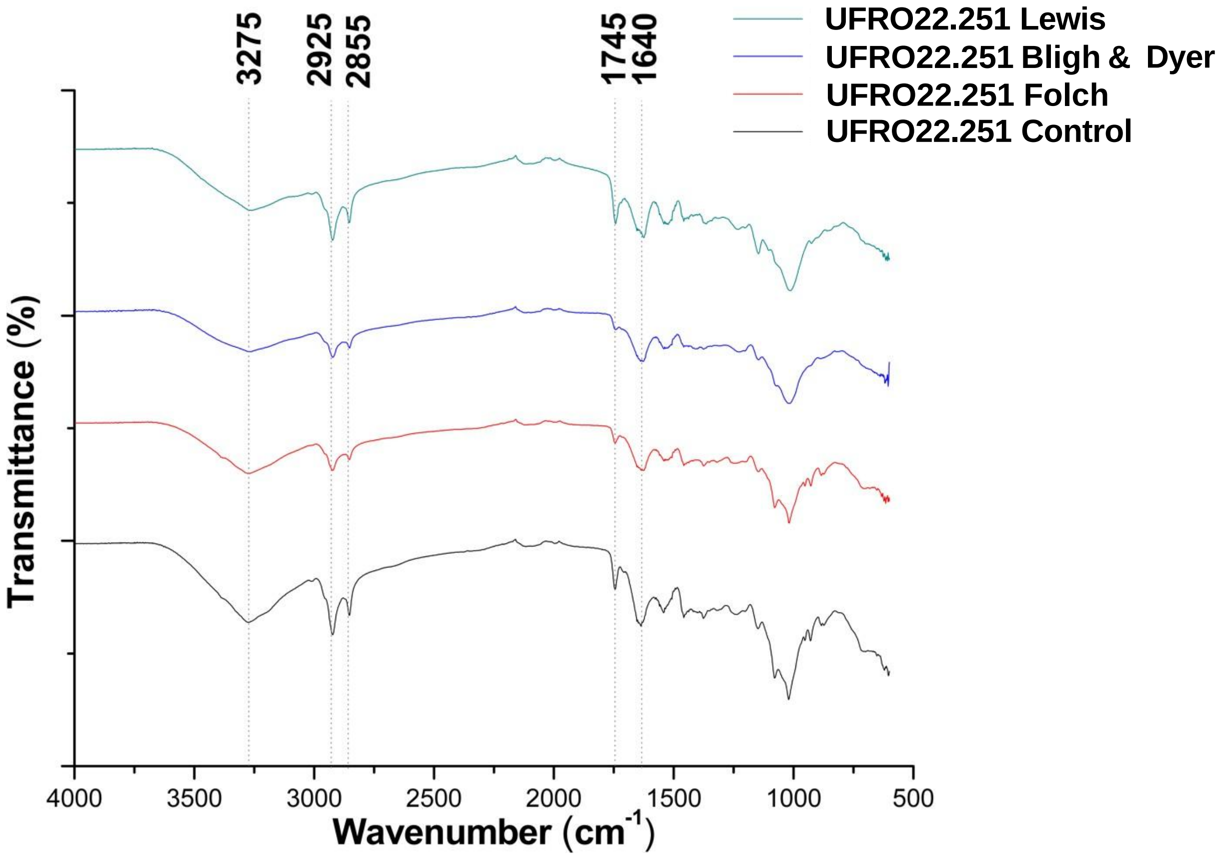

Figure S20. Infrared spectra for fungi biomass *Penicillium virgatum* (UFRO22.251), before (black line) and after extraction with Lewis (green line), Bligh & Dyer (blue line), Folch (red line) methods. Control is presented for each strain.

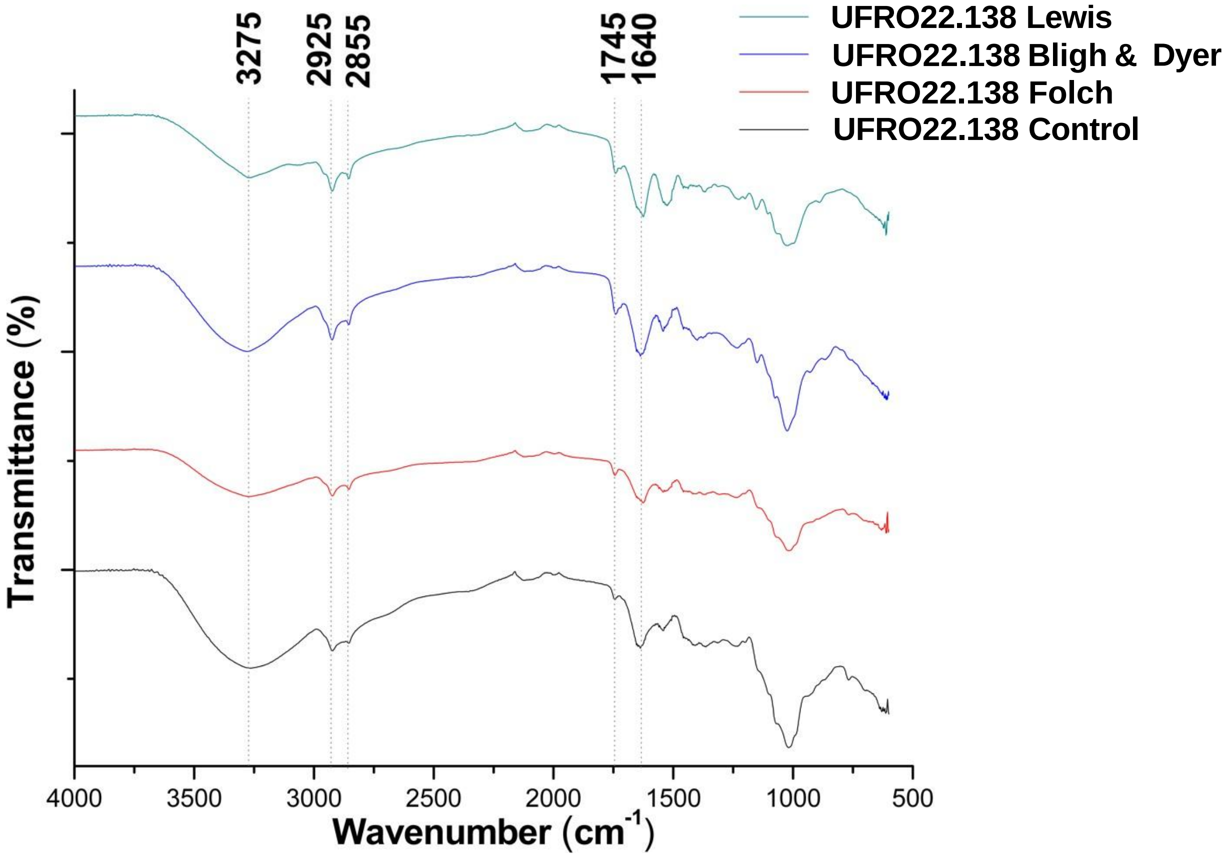

Figure S21. Infrared spectra for fungi biomass *Pseudogymnoascus pannorum* (UFRO22.138), before (black line) and after extraction with Lewis (green line), Bligh & Dyer (blue line), Folch (red line) methods. Control is presented for each strain.

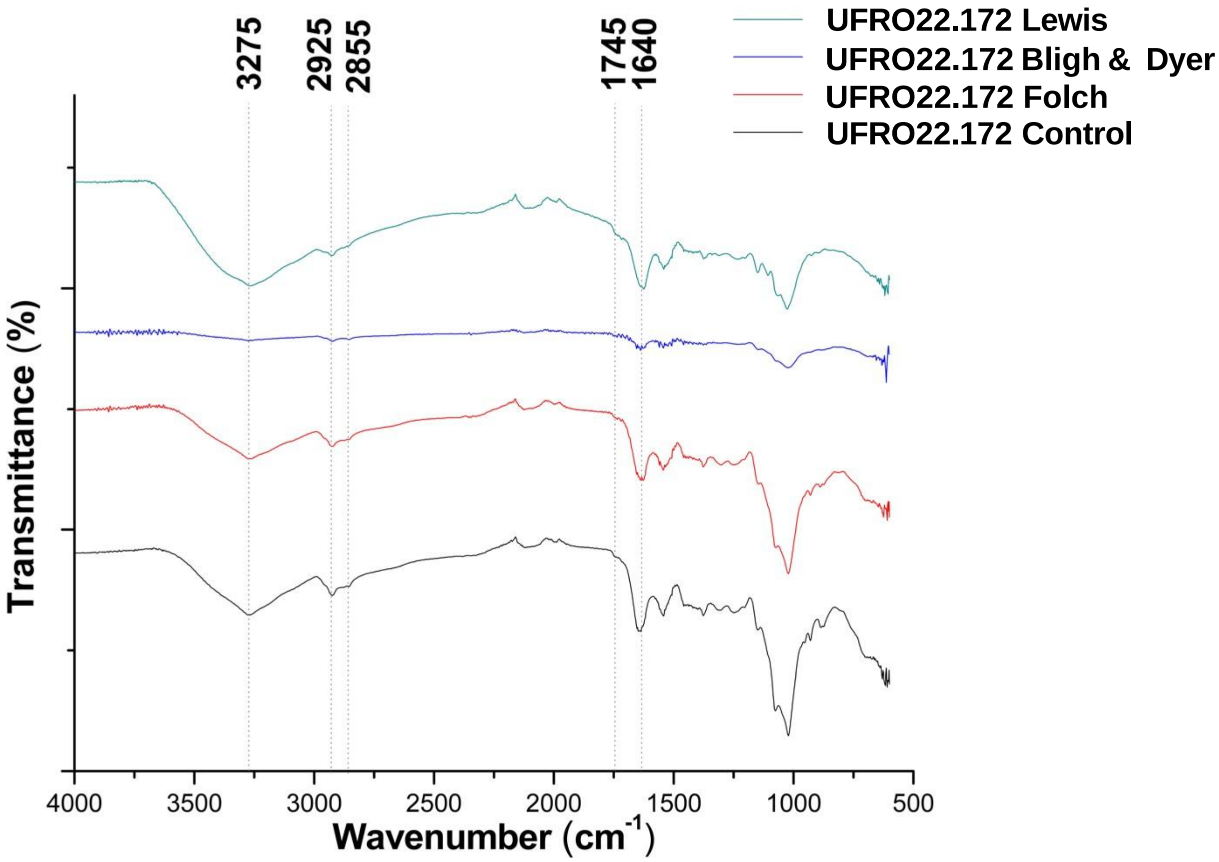

Figure S22. Infrared spectra for fungi biomass *Pseudogymnoascus pannorum* (UFRO22.172), before (black line) and after extraction with Lewis (green line), Bligh & Dyer (blue line), Folch (red line) methods. Control is presented for each strain.

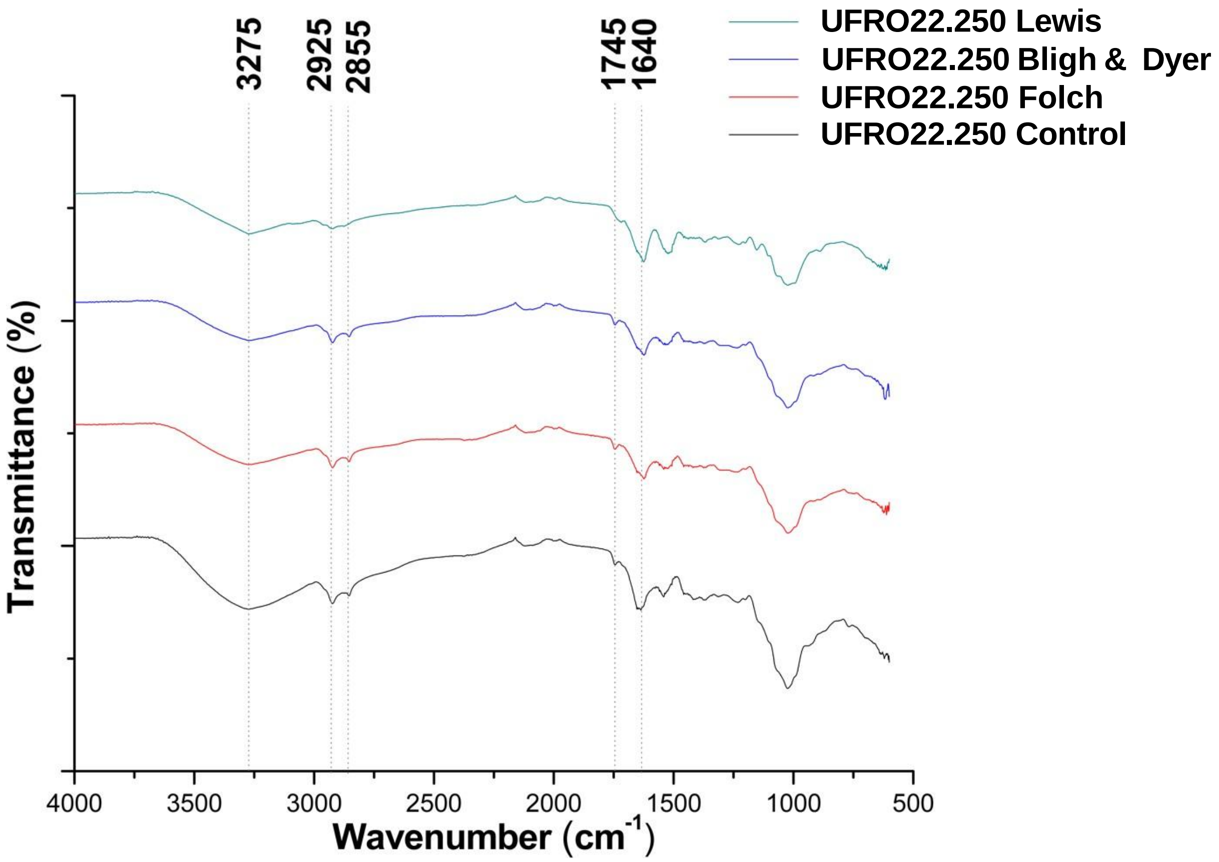

Figure S23. Infrared spectra for fungi biomass *Pseudogymnoascus pannorum* (UFRO22.250), before (black line) and after extraction with Lewis (green line), Bligh & Dyer (blue line), Folch (red line) methods. Control is presented for each strain.

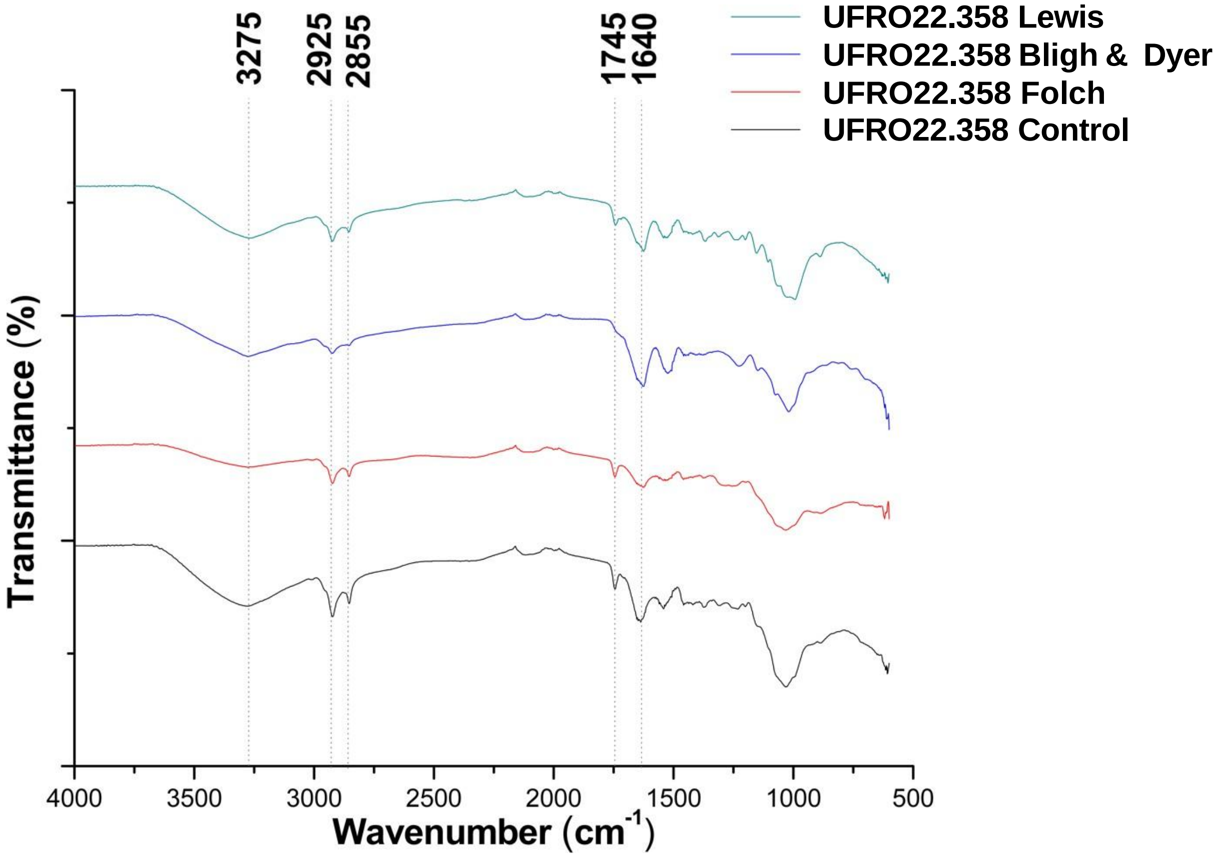

Figure S24. Infrared spectra for fungi biomass *Pseudogymnoascus pannorum* (UFRO22.358), before (black line) and after extraction with Lewis (green line), Bligh & Dyer (blue line), Folch (red line) methods. Control is presented for each strain.

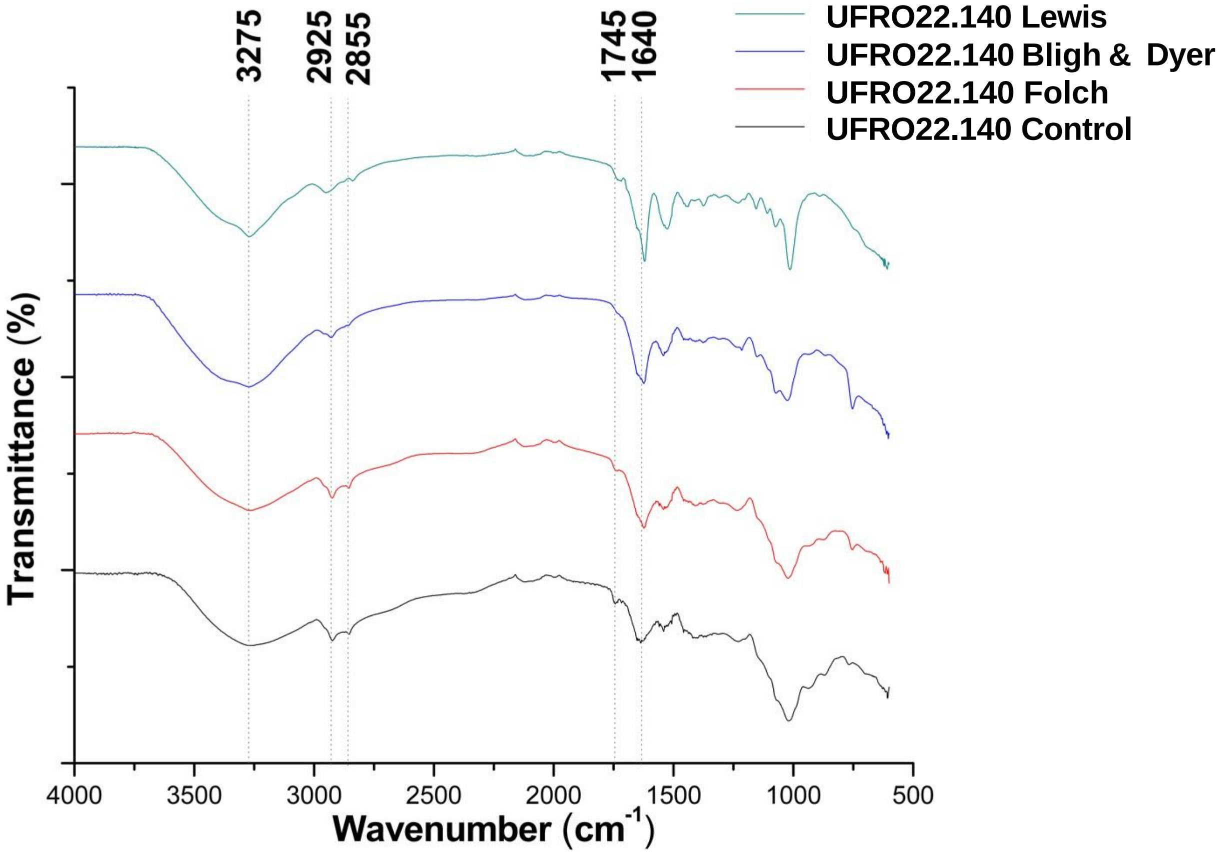

Figure S25. Infrared spectra for fungi biomass *Talaromyces acaricola* sect. *Islandici* (UFRO22.140), before (black line) and after extraction with Lewis (green line), Bligh & Dyer (blue line), Folch (red line) methods. Control is presented for each strain.

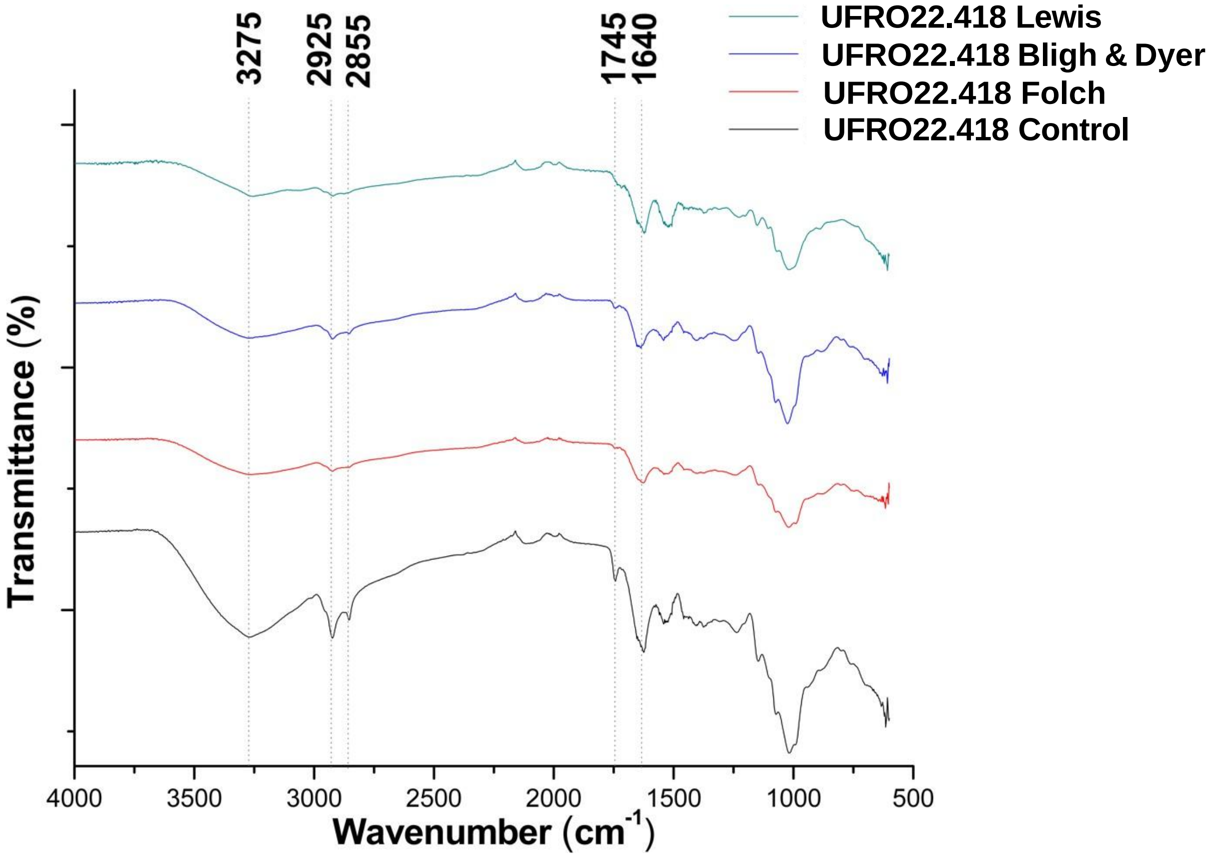

Figure S26. Infrared spectra for fungi biomass Melanommataceae family (UFRO22.418), before (black line) and after extraction with Lewis (green line), Bligh & Dyer (blue line), Folch (red line) methods. Control is presented for each strain.

Table S1. Effectiveness of lipid extraction among each fungal strain.

UFRO accesses	Taxonomy	Bligh & Dye	Folch	Lewis
**UFRO22.77**	*Botrytis cinerea*	12.93%	21.56%	14.88%
**UFRO22.262**	*Botrytis cinerea*	0.96%	1.13%	3.28%
**UFRO22.551**	*Cladosporium herbarum* complex herbarum	0.84%	2.01%	4.63%
**UFRO22.307**	*Cladosporium perangustum* complex cladosporioides	2.15%	2.41%	3.45%
**UFRO22.53**	*Cladosporium varians* complex cladosporioides	1.26%	2.38%	5.17%
**UFRO22.226**	*Cylindrobasidium eucalypti*	1.90%	5.45%	7.15%
**UFRO22.73**	*Mortierella antartica*	8.66%	28.15%	12.60%
**UFRO22.40**	*Mortierella gamsii*	15.79%	27.88%	17.53%
**UFRO22.317**	*Mortierella globulifera*	2.24%	4.95%	5.31%
**UFRO22.261**	*Mortierella truficola*	2.79%	9.59%	6.56%
**UFRO22.569**	*Penicillium miczynskii*	1.23%	6.38%	5.75%
**UFRO22.251**	*Penicillium virgatum*	4.57%	9.05%	6.28%
**UFRO22.138**	*Pseudogymnoascus pannorum*	2.94%	4.25%	7.35%
**UFRO22.172**	*Pseudogymnoascus pannorum*	2.36%	6.11%	9.19%
**UFRO22.250**	*Pseudogymnoascus pannorum*	1.57%	4.07%	7.13%
**UFRO22.358**	*Pseudogymnoascus pannorum*	2.03%	8.27%	6.28%
**UFRO22.140**	*Talaromyces acaricola* sect. *Islandici*	1.65%	5.38%	4.52%
**UFRO22.418**	Melanommataceae family	3.05%	6.51%	8.64%

Table S2. Average method quantification of each fatty acids obtained with Bligh & Dyer, Folch, and Lewis

Fatty acid	Bligh & Dyer (mg)				Folch (mg)				Lewis (mg)			
C12:0	0.09	±	0.13	c	0.13	±	0.29	bc	0.43	±	1.2	a
C14:0	0.08	±	0.1	c	0.1	±	0.13	b	0.29	±	0.2	a
C15:0	0.05	±	0.06	c	0.05	±	0.06	bc	0.13	±	0.07	a
C16:0	0.16	±	0.2	c	0.19	±	0.27	b	0.84	±	0.44	a
C18:0	0.37	±	0.66	c	0.47	±	1.02	bc	1.26	±	1.19	a
C18:1	0.69	±	0.92	c	0.89	±	1.35	b	3.07	±	1.98	a
C18:2	0.31	±	0.32	c	0.38	±	0.41	b	2.49	±	1.95	a
α-C18:3	0.06	±	0.07	c	0.07	±	0.09	bc	0.38	±	0.32	a
γ-C18:3	0.08	±	0.17	a	0.11	±	0.22	a	0.24	±	0.4	a
C20:3	0.06	±	0.11	c	0.07	±	0.16	bc	0.26	±	0.1	a
C20:4	0.08	±	0.18	a	0.16	±	0.4	a	0.28	±	0.5	a
C20:5	0.04	±	0.08	a	0.05	±	0.09	a	0.08	±	0.12	a
C22:0	0.07	±	0.1	c	0.07	±	0.11	bc	0.15	±	0.11	a
C24:0	0.07	±	0.1	c	0.08	±	0.14	bc	0.21	±	0.1	a
C24:1	0.05	±	0.11	a	0.06	±	0.12	a	0.1	±	0.15	a

[a]Data are shown as mean ± standard deviation (n = 18)

Table S3. Fatty acids yield quantified in all extracts, obtained by the Bligh & Dyer method.

UFRO accesses	Taxonomy	C16:0	C18:0	C18:1	C18:2	α-C18:3	C12:0	C14:0	C15:0	γ-C18:3	C22:0	C20:3	C20:4	C24:0	C20:5	C24:1
UFRO22.262	*Botrytis cinerea*	1.6%	1.8%	2.0%	3.2%	2.1%	1.1%	1.2%	1.1%	Tr	Tr	Tr	Tr	Tr	Tr	Tr
UFRO22.77	*Botrytis cinerea*	0.8%	3.3%	5.4%	0.8%	Tr	0.4%	0.6%	0.5%	0.8%	0.5%	0.7%	0.7%	0.6%	0.6%	0.7%
UFRO22.307	*Cladosporium herbarum* complex *herbarum*	0.4%	0.6%	1.1%	1.4%	0.8%	0.5%	0.5%	Tr	Tr	Tr	Tr	Tr	Tr	Tr	Tr
UFRO22.53	*Cladosporium perangustum* complex cladosporioides	1.1%	1.3%	2.7%	3.1%	1.5%	0.8%	0.9%	Tr	Tr	Tr	Tr	Tr	Tr	Tr	Tr
UFRO22.551	*Cladosporium varians* complex cladosporioides	1.5%	2.0%	2.5%	3.0%	2.0%	1.3%	Tr	Tr	Tr	Tr	Tr	Tr	Tr	Tr	Tr
UFRO22.226	*Cylindrobasidium eucalypti*	0.8%	0.7%	2.7%	3.0%	1.5%	11.5%	1.3%	0.5%	1.1%	0.5%	0.9%	1.4%	0.7%	0.8%	0.9%
UFRO22.73	*Mortierella antartica*	2.5%	5.2%	9.7%	2.0%	Tr	0.6%	1.2%	0.8%	2.2%	1.0%	1.1%	1.2%	1.1%	0.9%	1.1%
UFRO22.40	*Mortierella gamsii*	1.8%	6.6%	8.0%	1.5%	Tr	0.4%	0.9%	0.3%	1.3%	0.8%	0.9%	1.8%	1.0%	0.6%	0.9%
UFRO22.317	*Mortierella globulifera*	1.0%	1.6%	6.1%	1.7%	Tr	0.5%	0.8%	0.6%	1.6%	0.5%	0.9%	2.1%	0.6%	0.7%	0.8%
UFRO22.261	*Mortierella truficola*	0.6%	0.6%	3.1%	0.7%	Tr	0.4%	0.5%	0.4%	1.1%	0.4%	0.7%	1.7%	0.4%	0.6%	0.6%
UFRO22.569	*Penicillium miczynskii*	2.0%	2.5%	5.8%	11.5%	3.1%	0.9%	0.9%	0.8%	Tr	Tr	Tr	Tr	0.9%	Tr	Tr
UFRO22.251	*Penicillium virgatum*	1.9%	3.3%	6.6%	4.0%	1.9%	1.1%	1.1%	1.1%	Tr	1.2%	Tr	Tr	Tr	Tr	Tr
UFRO22.358	*Pseudogymnoascus pannorum*	1.2%	1.3%	5.5%	3.8%	1.0%	0.5%	0.6%	0.5%	Tr	0.5%	Tr	Tr	0.5%	Tr	Tr
UFRO22.138	*Pseudogymnoascus pannorum*	2.8%	3.5%	10.1 %	7.6%	3.0%	1.8%	1.8%	Tr	Tr	1.9%	Tr	Tr	1.9%	Tr	Tr
UFRO22.250	*Pseudogymnoascus pannorum*	2.1%	2.5%	5.4%	3.1%	1.2%	0.7%	0.8%	0.7%	Tr	0.7%	Tr	Tr	0.7%	Tr	Tr
UFRO22.172	*Pseudogymnoascus pannorum*	1.3%	2.1%	4.8%	6.8%	0.9%	0.6%	0.5%	0.5%	Tr	Tr	Tr	Tr	0.5%	Tr	Tr
UFRO22.140	*Talaromyces acaricola* sect. *Islandici*	1.1%	1.6%	2.7%	3.6%	Tr	0.6%	0.7%	0.6%	Tr	Tr	Tr	Tr	0.7%	Tr	Tr
UFRO22.418	Melanommataceae family	4.1%	4.9%	24.1%	17.8%	2.4%	0.4%	0.4%	0.3%	Tr	0.4%	Tr	Tr	0.4%	Tr	Tr

Tr: Trace

Table S4. Fatty acids yield quantified in all extracts, obtained by the Folch method.

UFRO accesses	Taxonomy	C16:0	C18:0	C18:1	C18:2	α-C18:3	C12:0	C14:0	C15:0	γ-C18:3	C22:0	C20:3	C20:4	C24:0	C20:5	C24:1
UFRO22.262	*Botrytis cinerea*	1.1%	1.0%	1.8%	4.3%	2.3%	0.9%	1.0%	0.9%	Tr	Tr	Tr	Tr	Tr	Tr	Tr
UFRO22.77	*Botrytis cinerea*	0.9%	3.7%	6.0%	0.7%	Tr	0.3%	0.5%	0.3%	0.7%	0.4%	0.5%	0.5%	0.5%	0.4%	0.5%
UFRO22.307	*Cladosporium herbarum* complex *herbarum*	0.6%	0.7%	1.7%	2.1%	0.8%	0.5%	0.5%	Tr	Tr	Tr	Tr	Tr	Tr	Tr	Tr
UFRO22.53	*Cladosporium perangustum* complex cladosporioides	0.9%	1.0%	2.5%	3.2%	1.1%	0.5%	0.5%	Tr	Tr	Tr	Tr	Tr	Tr	Tr	Tr
UFRO22.551	*Cladosporium varians* complex cladosporioides	0.8%	1.1%	1.3%	1.8%	1.0%	0.5%	0.5%	Tr	Tr	Tr	Tr	Tr	Tr	Tr	Tr
UFRO22.226	*Cylindrobasidium eucalypti*	0.5%	0.4%	2.1%	2.4%	0.9%	9.4%	0.8%	0.2%	0.7%	0.2%	0.4%	0.9%	0.3%	0.3%	0.3%
UFRO22.73	*Mortierella antartica*	0.1%	0.2%	0.3%	0.1%	Tr	Tr	0.1%	Tr	0.1%	Tr	0.1%	0.1%	0.1%	0.1%	0.1%
UFRO22.40	*Mortierella gamsii*	1.5%	5.8%	7.0%	1.3%	Tr	0.3%	0.7%	0.2%	1.2%	0.6%	0.9%	2.3%	0.8%	0.4%	0.6%
UFRO22.317	*Mortierella globulifera*	0.6%	1.0%	3.7%	1.3%	Tr	0.2%	0.4%	0.3%	0.8%	0.2%	0.5%	1.1%	0.3%	0.4%	0.4%
UFRO22.261	*Mortierella truficola*	0.8%	0.9%	5.1%	1.1%	Tr	0.5%	0.7%	0.5%	1.7%	0.5%	1.1%	2.8%	0.6%	0.8%	0.9%
UFRO22.569	*Penicillium miczynskii*	0.5%	0.6%	1.2%	2.8%	0.7%	0.2%	0.2%	0.2%	Tr	Tr	Tr	Tr	0.2%	Tr	Tr
UFRO22.251	*Penicillium virgatum*	0.8%	1.5%	3.0%	1.6%	0.3%	0.1%	0.1%	0.1%	Tr	0.1%	Tr	Tr	0.1%	Tr	Tr
UFRO22.358	*Pseudogymnoascus pannorum*	1.0%	1.1%	4.3%	3.0%	1.1%	0.7%	0.7%	0.6%	Tr	0.7%	Tr	Tr	Tr	Tr	Tr
UFRO22.138	*Pseudogymnoascus pannorum*	1.0%	1.2%	3.9%	2.6%	0.6%	0.3%	0.3%	0.2%	Tr	0.3%	Tr	Tr	0.3%	Tr	Tr
UFRO22.250	*Pseudogymnoascus pannorum*	0.5%	0.6%	1.7%	1.0%	0.4%	0.6%	0.3%	0.3%	0.4%	0.3%	Tr	Tr	0.3%	Tr	Tr
UFRO22.172	*Pseudogymnoascus pannorum*	0.2%	0.3%	0.5%	0.8%	0.3%	0.2%	0.2%	Tr	Tr	Tr	Tr	Tr	0.2%	Tr	Tr
UFRO22.140	*Talaromyces acaricola* sect. *Islandici*	0.6%	1.0%	2.3%	3.2%	0.0%	0.2%	0.2%	0.2%	Tr	Tr	Tr	Tr	0.3%	Tr	Tr
UFRO22.418	Melanommataceae family	2.9%	3.1%	14.4%	11.0%	2.2%	0.8%	0.9%	0.8%	Tr	0.9%	Tr	Tr	0.9%	Tr	Tr

Tr: Trace

Table S5. Fatty acids yield quantified in all extracts, obtained by the Lewis method.

UFRO accesses	Taxonomy	C16:0	C18:0	C18:1	C18:2	α-C18:3	C12:0	C14:0	C15:0	γ-C18:3	C22:0	C20:3	C20:4	C24:0	C20:5	C24:1
UFRO22.262	*Botrytis cinerea*	4.0%	3.4%	4.2%	18.3%	6.6%	1.6%	2.3%	1.6%	Tr	Tr	2.3%	Tr	1.6%	Tr	Tr
UFRO22.77	*Botrytis cinerea*	1.5%	4.3%	6.7%	1.1%	Tr	0.5%	0.9%	0.4%	1.1%	0.5%	0.8%	1.0%	0.6%	0.7%	0.6%
UFRO22.307	*Cladosporium herbarum* complex *herbarum*	3.6%	4.3%	7.9%	16.3%	3.8%	1.5%	1.6%	Tr	Tr	Tr	2.2%	Tr	1.6%	Tr	Tr
UFRO22.53	*Cladosporium perangustum* complex cladosporioides	5.8%	6.0%	12.8%	16.9%	3.8%	1.0%	1.3%	Tr	Tr	Tr	1.5%	Tr	1.1%	Tr	Tr
UFRO22.551	*Cladosporium varians* complex cladosporioides	4.5%	5.8%	10.3%	22.0%	5.7%	1.1%	1.2%	Tr	Tr	Tr	1.6%	Tr	1.3%	Tr	Tr
UFRO22.226	*Cylindrobasidium eucalypti*	3.4%	2.9%	8.8%	13.7%	5.0%	29.0%	3.2%	0.8%	2.7%	0.8%	1.9%	3.5%	1.5%	1.4%	1.3%
UFRO22.73	*Mortierella antartica*	5.5%	10.1%	18.3%	3.6%	Tr	0.4%	1.9%	0.9%	3.8%	1.3%	1.2%	1.8%	1.4%	0.7%	1.0%
UFRO22.40	*Mortierella gamsii*	4.0%	11.6%	17.6%	1.8%	Tr	0.7%	2.0%	0.4%	1.8%	0.9%	1.1%	2.2%	1.1%	0.6%	1.0%
UFRO22.317	*Mortierella globulifera*	2.9%	4.0%	13.9%	3.8%	Tr	1.0%	2.4%	1.2%	3.6%	1.1%	2.3%	4.8%	1.4%	1.7%	1.8%
UFRO22.261	*Mortierella truficola*	3.6%	3.0%	21.2%	2.8%	Tr	0.8%	2.6%	0.9%	5.9%	0.8%	3.0%	11.1 %	1.3%	1.5%	1.7%
UFRO22.569	*Penicillium miczynskii*	5.2%	6.5%	12.5%	29.3%	6.0%	1.0%	1.0%	0.9%	Tr	0.9%	1.3%	Tr	1.1%	Tr	Tr
UFRO22.251	*Penicillium virgatum*	4.7%	8.2%	15.1%	14.2%	2.8%	0.8%	1.3%	0.9%	Tr	0.9%	1.2%	Tr	0.9%	Tr	Tr
UFRO22.358	*Pseudogymnoascus pannorum*	4.2%	4.2%	20.9%	13.8%	2.0%	0.8%	1.1%	0.8%	Tr	0.9%	1.2%	Tr	0.9%	Tr	Tr
UFRO22.138	*Pseudogymnoascus pannorum*	5.6%	6.1%	20.9%	14.7%	2.3%	0.7%	1.2%	0.7%	Tr	1.0%	1.0%	Tr	0.9%	Tr	Tr
UFRO22.250	*Pseudogymnoascus pannorum*	5.2%	4.9%	26.3%	14.2%	3.5%	0.7%	1.2%	0.7%	Tr	0.9%	1.0%	Tr	0.9%	Tr	Tr
UFRO22.172	*Pseudogymnoascus pannorum*	6.0%	8.5%	16.9%	36.3%	1.8%	0.6%	0.7%	0.7%	Tr	0.7%	0.8%	Tr	0.9%	Tr	Tr
UFRO22.140	*Talaromyces acaricola* sect. *Islandici*	6.1%	8.9%	18.6%	34.3%	Tr	1.2%	1.5%	1.2%	Tr	1.2%	1.7%	Tr	1.5%	Tr	Tr
UFRO22.418	Melanommataceae family	5.2%	4.9%	25.5%	20.3%	3.5%	0.6%	0.9%	0.6%	Tr	0.7%	0.9%	Tr	0.7%	Tr	Tr

Tr: Trace
